# Kynurenine Pathway Metabolites as Potential Clinical Biomarkers in Coronary Artery Disease

**DOI:** 10.3389/fimmu.2021.768560

**Published:** 2022-02-08

**Authors:** Renáta Gáspár, Dóra Halmi, Virág Demján, Róbert Berkecz, Márton Pipicz, Tamás Csont

**Affiliations:** ^1^Metabolic Diseases and Cell Signaling Research Group (MEDICS), Department of Biochemistry, University of Szeged Albert Szent-Györgyi Medical School, Szeged, Hungary; ^2^Interdisciplinary Centre of Excellence, University of Szeged, Szeged, Hungary; ^3^Institute of Pharmaceutical Analysis, Faculty of Pharmacy, University of Szeged, Szeged, Hungary

**Keywords:** tryptophan, kynurenic acid, personalized medicine, ischemic heart disease, liquid chromatography, mass spectrometry, prediction, IDO activity/detection

## Abstract

Coronary artery disease (CAD) is one of the leading cause of mortality worldwide. Several risk factors including unhealthy lifestyle, genetic background, obesity, diabetes, hypercholesterolemia, hypertension, smoking, age, etc. contribute to the development of coronary atherosclerosis and subsequent coronary artery disease. Inflammation plays an important role in coronary artery disease development and progression. Pro-inflammatory signals promote the degradation of tryptophan *via* the kynurenine pathway resulting in the formation of several immunomodulatory metabolites. An unbalanced kynurenic pathway has been implicated in the pathomechanisms of various diseases including CAD. Significant improvements in detection methods in the last decades may allow simultaneous measurement of multiple metabolites of the kynurenine pathway and such a thorough analysis of the kynurenine pathway may be a valuable tool for risk stratification and determination of CAD prognosis. Nevertheless, imbalance in the activities of different branches of the kynurenine pathway may require careful interpretation. In this review, we aim to summarize clinical evidence supporting a possible use of kynurenine pathway metabolites as clinical biomarkers in various manifestations of CAD.

## 1 Introduction

Coronary artery disease (CAD), sometimes called ischemic heart disease or coronary heart disease, is one of the leading cause of disability and death worldwide. In 2017, CAD affected around 126 million individuals and caused 9 million deaths globally (1). CAD represents a group of pathologically related conditions characterized by atherosclerosis of cardiac arteries and a potential functional complication of coronary circulation. The inadequate perfusion of the myocardium results in discrepancy between oxygen demand and supply, reduced availability of nutrients and incomplete removal of metabolic end products (2). CAD manifests as either acute coronary syndrome (ACS) or chronic coronary syndrome (3). Sudden impairment of myocardial blood supply in ACS may present in the form of unstable angina or myocardial infarction, and the severity ranges from a chest pain to cardiac arrest (4). According to the latest ESC guidelines, chronic coronary syndrome includes i) stable coronary artery diseases such as stable angina, coronary spasm or microvascular angina, ii) new onset heart failure or left ventricular dysfunction with suspected CAD and iii) stabilized conditions after recent revascularization or within the 1^st^ year after ACS events (3). All these conditions share similar pathophysiology in which inflammation plays a role (5) and many inflammatory biomarkers (e.g. C-reactive protein (CRP), interleukin(IL)-6, myeloperoxidase, soluble CD40 ligand, etc.) may have a potential role for predicting CAD or assessing the severity of CAD (6). Pro-inflammatory signals have been reported to facilitate tryptophan metabolism through the kynurenine pathway, thereby leading to the formation of several immunomodulatory metabolites (7). An unbalanced kynurenine pathway (KP) has been implicated in the pathomechanisms of various diseases including CAD, indicating a potential diagnostic or predictive role for KP metabolites. Therefore, here we review the literature on the potential use of KP metabolites as clinical biomarkers in CAD and evaluate the currently available detection methods.

## 2 CAD, Atherosclerosis, and Inflammation

The most important mechanism in the background of CAD is atherosclerotic plaque accumulation in epicardial coronary arteries (8), which is influenced by various genetic and environmental factors, lifestyle, and both pharmacological and invasive interventions. Inflammation plays a crucial role in all stages of atherosclerotic plaque formation. Smoking, lack of physical activity, unhealthy diet and certain health problems including but not limited to diabetes mellitus, obesity, metabolic syndrome, hypertension, hypercholesterolemia, or homocystinuria have been found to be the most important modifiable risk factors of CAD (9), contributing to endothelial dysfunction. Endothelial cell activation during the initiation of atherogenic processes leads to expression and release of pro-inflammatory factors, chemoattractant and adhesion molecules, which results in leukocyte and monocyte infiltration of arterial walls, leading to inflammation (10). Inflammation has a fundamental role during late-stage atherosclerosis as well: it enhances the local accumulation of macrophages that are responsible for the weakening of the fibrous cap of plaques by releasing collagen-degrading matrix metalloproteinases (11). Destabilization of the cap increases the risk of plaque rupture, which suggests that inflammation has an important role not only during atherogenesis, but during the development of ACS as well. This statement was supported by several independent studies which indicated the predictive value of pro-inflammatory molecules in blood serum, such as IL-6, tumor necrosis factor-α or CRP for the incidence of cardiovascular diseases (12–14). It was also reported that adaptive immunity alterations including failure to control the activation of aggressive T-cells might be associated with worse outcome in ACS patients and can be rarely identified in patients with stable coronary disease, and were never seen in healthy controls (15). This suggests that not only inflammatory, but other aspects of immune functions might also have important roles during the development of atherosclerosis and CAD. The fact that certain autoimmune diseases, such as rheumatoid arthritis and systemic lupus erythematosus have been associated with higher prevalence of atherosclerosis, hypertension, and increased cardiovascular mortality, supports this statement (16, 17). More detailed understanding of these mechanisms might help the identification of new biomarkers and potential therapeutic targets which may support both the follow-up and treatment of patients with cardiovascular diseases.

## 3 The Kynurenine Pathway

The physiological role of KP, the major route of tryptophan degradation, in the heart and vasculature is not completely clear yet. Under normal conditions the pathway plays an important role in generating nicotinic acid (vitamin B3) and therefore contributing to cellular energetic homeostasis in form of nicotinamide adenine dinucleotide (NAD+) (18). NAD+ is a common redox cofactor in various biological processes, including calcium homeostasis, energy metabolism, mitochondrial functions, and antioxidant/prooxidant balance which are particularly relevant in the heart and vascular system (19). Although the exact physiological role of other members of KP is unknown in the cardiovascular system, certain metabolites may contribute to vascular tone regulation, especially during inflammation (20).

Increasing number of studies indicates that KP is altered in cardiovascular diseases; however, it is still unclear whether or not the endogenous kynurenines are directly involved in the initiation or progression of CAD (21). The importance of the KP in cardiovascular diseases may include the patho-mechanistic involvement in cardiovascular risk factors, such as hypertension, diabetes mellitus, dyslipidemia and obesity, as well as in vascular inflammation and atherosclerosis in CAD (21, 22).

**Figure 1:**
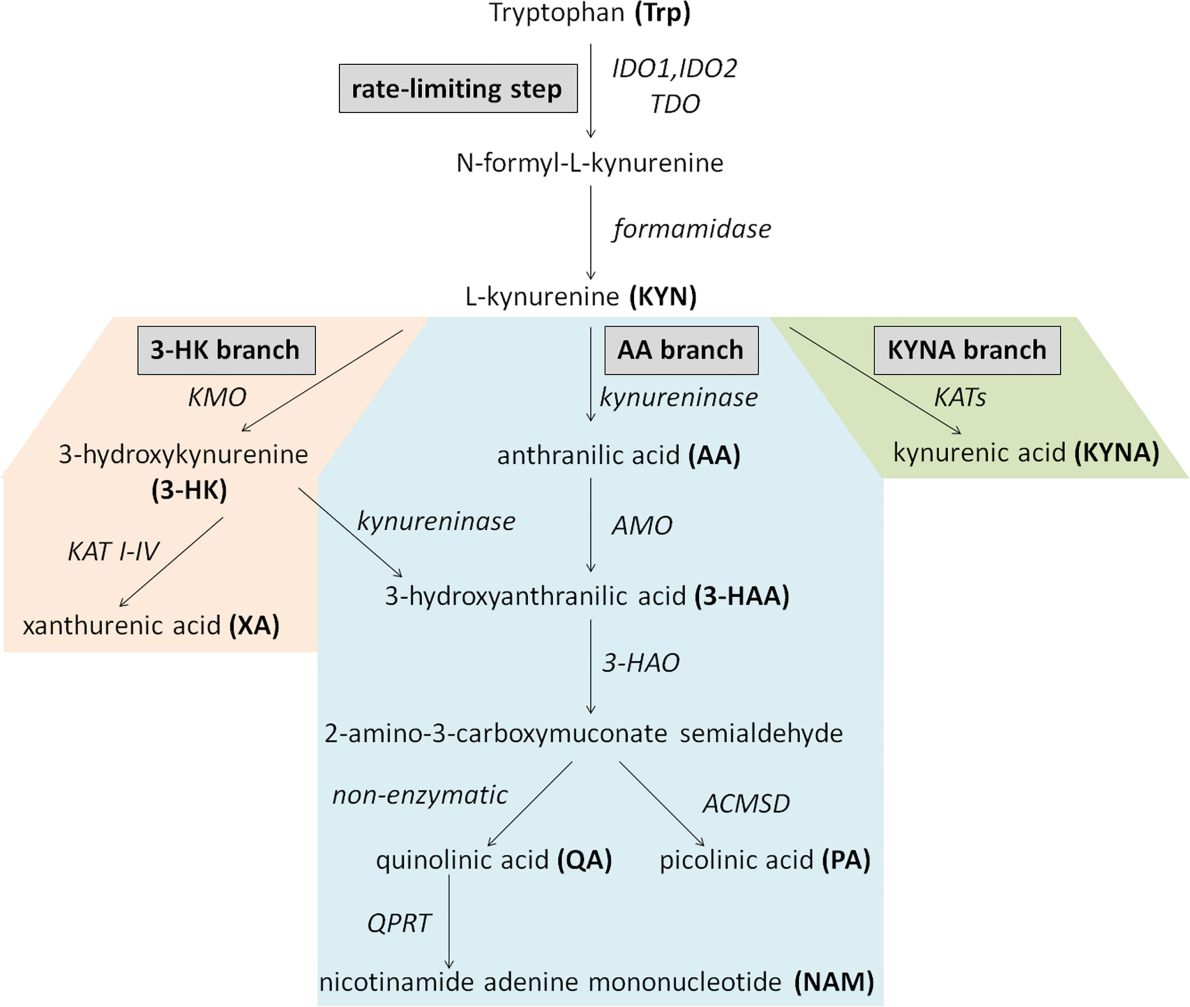
Schematic overview of the kynurenine pathway. Enzymes are indicated in italics. AA, anthranilic acid; ACMSD, aminocarboxymuconate-semialdehyde-decarboxylase; AMO, anthranilate 3-monooxygenase; 3-HAA, 3-hydroxyanthranilic acid; 3-HAO, 3-hydroxyanthranilate 3,4-dioxygenase; 3-HK, 3-hydroxykynurenine; IDO1 and or, IDO2, indoleamine 2,3-dioxygenase-1 and -2; KATs, kynurenine aminotransferase enzymes; KMO, kynurenine monooxygenase; KYN, kynurenine; KYNA, kynurenic acid; NAM, nicotinamide adenine mononucleotide; PA, picolinic acid; QA, quinolinic acid; QPRT, quinolinate phosphoribosyltransferase; TDO, tryptophan-2,3-dioxygenase; Trp, Tryptophan; XA, xanthurenic acid.

In humans, approximately 95% of catabolized tryptophan (Trp) is converted to immunomodulating compounds, collectively termed kynurenines (**Figure 1**). The conversion of Trp to N-formyl-L-kynurenine is the rate-limiting first step of the KP which can be catalyzed by three different enzymes: indoleamine 2,3-dioxygenase-1 and -2 (IDO1, IDO2) or tryptophan-2,3-dioxygenase (TDO). While TDO functions mainly in the liver, controlling the concentration of Trp in the circulation, IDO enzymes are responsible for the initiation of KP in extrahepatic tissues to produce large number of metabolites involved in various physiological and pathophysiological processes (23). N-formyl-L-kynurenine is then converted to L-kynurenine (KYN) by formamidase. Kynurenine/tryptophan ratio (KYN/Trp ratio, KTR) is considered as an indicator of rate-limiting IDO/TDO activity. KYN is the central intermediate of the pathway, which can be metabolized further by three enzymes, initiating the three main branches of KP (**Figure 1**). Kynurenine monooxygenase (KMO) catalyzes the production of 3-hydroxykynurenine (3-HK), while kynureninase contributes to anthranilic acid (AA) formation. Both 3-HK and AA can be converted to hydroxyanthranilic acid (3-HAA), then to 2-amino-3-carboxymuconate semialdehyde, precursor of quinolinic acid (QA) and picolinic acid (PA). Under physiological condition, the majority of KYN is metabolized through these branches to produce NAD^+^ from QA (18). 3-HK can be converted to xanthurenic acid (XA) as well. The third main route of KYN breakdown is the formation of kynurenic acid (KYNA) *via* kynurenine aminotransferase enzymes (KAT I-IV) (**Figure 1**) (23).

The available literature data is limited regarding the activities of different KP branches (i.e. 3-HK, AA, KYNA branch) and probably their proportions are different among tissues and cell types, both under physiological conditions and diseases. In patients without heart failure and CAD who underwent coronary angiography the following ratios were measured in blood: 3-HK/KYN was 1.9%, AA/KYN was 0.9%, KYNA/KYN was 2.8% and 3 HAA/KYN was 2.0% (24). In human atherosclerotic arteries RNA transcripts of IDO, TDO, KMO and kynureninase enzymes were increased, while the levels of KATI-II were decreased versus controls, which indicates a deviation in KP branches (25). In the brain under physiological conditions the synthesis of 3-HK and KYNA is approximately evenly proportioned (26); however, in inflammatory conditions it shifts in the direction of 3-HK synthesis (27).

Enzymes of KP are expressed in wide variety of organs/tissues/cells. Regarding the cardiovascular system, cardiomyocytes, endothelial cells, fibroblasts, smooth muscle cells and immune cells are relevant. Several enzymes are expressed in these cells, for detailed information see **Supplement Table 1**. Table shows cell type-specific RNA expression of enzymes involved in KP from healthy human tissues (**Supplement Table 1**) according to transcriptomics datasets of www.proteinatlas.org (28). The rate-limiting IDO expression is not detected in cardiomyocytes, fibroblasts and dendritic cells, and expression is low in endothelial, smooth muscle and T cells, and high in macrophages and monocytes (**Supplement Table 1**) in healthy persons. However, the expression is induced under cardiovascular disease related conditions in most of the cell types (29–35) (**Table 1**). It suggests that the intensity of IDO expression is not constant, and seems to be enhanced in CAD.

**Table 1:** IDO expression under cardiovascular disease related conditions.

Cell type	Species	Source	Condition	Expression	Ref.
cardiomyocyte	neonatal rat	heart left ventricle	*in vitro* cardiomyocyte hypertrophy induced by treatment with angiotensin II, isoproterenol, phenylephrine	mRNA and protein	(34)
cardiac endothelial cells	mouse	heart left ventricle	1 day after *in vivo* myocardial infarction	mRNA and IDO activity assessed by measurement of KTR	(35)
vascular endothelial cells	human	aorta	induction with IFN-γ	protein	(31)
cardiac myofibroblasts	human	heart ventricles	induction with IFN-γ	protein	(33)
cardiac/stem progenitor cells	human	heart right atria appendage myocardial tissue	induction with IFN-γ	protein	(32)
serum	human	venous blood	patients in whom CAD was suspected, and underwent coronary angiogram	IDO activity assessed by measurement of KTR	(36)
aortic smooth muscle cells	human	aorta	induction with IFN-γ	IDO activity: paper chromatography	(30)
monocytes, macrophages	human	blood buffy coat	induction with IFN-γ	protein	(31)
dendritic cell	human	blood buffy coat	induction with IFN-γ	mRNA	(29)

Table shows that those cells which are relevant in coronary artery diseases (CAD) express indoleamine 2,3-dioxygenase (IDO) in response to cardiovascular pathology related stimuli. KTR, kynurenine/tryptophan ratio.

Members of KP exert effects on the same, kynurenine-producing cell (cis-action) (37) and distinct cells (trans-action) (38) mainly in receptor-dependent manner. For instance, KYN activates aryl hydrocarbon receptor (39). KYNA is an antagonist of glutamate receptors (e.g. NMDA, AMPA, kainate) and 7-nicotinic acetylcholine receptors, and agonist of aryl hydrocarbon receptor and GPR35 (40). QA activates glutamate receptors and may exert receptor-independent intracellular action as well. As a result of KP activation, Trp depletion is sensed by amino-acid sensors (e.g. GCN2, mTOR), leading to cellular changes (38).

Considerable amount of data supports the fundamental immunomodulatory role of KP. IDO is expressed by various immune cells and contributes to regulation of immune responses through several mechanisms, including modulation of signaling pathways and production of immunologically active KP metabolites, such as KYN, 3-HAA and KYNA (23). IDO is expressed in mainly dendritic cells, monocytes and macrophages (41), contributing to majority production of kynurenine metabolites. KYN have been found to reduce the activity of natural killer cells, dendritic cells, macrophages, monocytes and proliferation of T-lymphocytes (42). 3-HAA was found to directly inhibit the activation of dendritic cells, while KYNA provides anti-inflammatory and immunosuppressive functions *via* attenuation of pro-inflammatory cytokine production (43, 44).

It has been revealed that kynurenine metabolism becomes up-regulated through activation of IDO in response to inflammatory signals, from which IFN-γ is thought to be the main IDO activator (7). IDO is also activated by other inflammatory stimuli like lipopolysaccharide, IL-1, TNF, soluble CTLA4–immunoglobulin fusion protein, IFN-α IFN-β as well. Anti-inflammatory cytokines such as IL-4, IL-10, and TGFβ inhibit IFN-γ-induced IDO. In addition, CD40 ligation and nitric-oxide (NO) inhibits IDO activity (45, 46). Therefore, regulation of IDO expression is complex (45), the balance between pro- and anti-inflammatory signals determines the activity of IDO and KP (46). Although most cell types express IFN receptors, IFNs induce IDO considerably greater in few cell types like dendritic cells, macrophages or vascular smooth muscle cells than endothelial cells or Treg cells (47, 48).

In the cardiovascular system, endothelial cells synthetize large quantities of kynurenines, especially KYNA, the synthesis of which can be altered by the ionic milieu, oxygen, and nutrient supply (49). It is well-known that pro-inflammatory cytokines, such as IDO activator IFN-γ are important contributors of atherogenesis (50). The IFN-γ can be produced by resident cells (mainly by T-lymphocytes and macrophages) of atherosclerotic plaques (50), and extends the activation of macrophages leading to increased IDO1 activation and enhanced production of Trp metabolites (51). Additionally, recent evidence supports the key role for the KP in the regulation of inflammation and tolerance mechanism linked to atherosclerosis, thus IDO1 emerges as a key atheroprotective enzyme promoting immune homeostasis (52).

In conclusion, KP might be associated with pathogenesis of CAD by modulation of inflammatory processes as well. The potential involvement of the KP in CAD suggests that kynurenines might be utilized as biomarkers of CAD in the future with substantial diagnostic, predictive, prognostic and monitoring values.

## 4 Kynurenine Pathway Metabolites as Potential Clinical Biomarkers in CAD

### 4.1 Methods Available for the Detection of Kynurenine Pathway Metabolites: Clinical Relevance

The biologically active metabolites of KP might be used as measurable biomarkers for different pathological conditions. In the field of neurology, decreased Trp metabolites were reported in patients with migraine without aura in the interictal period (53), and KP metabolites were suggested to be promising biomarkers for amyotrophic lateral sclerosis (54). In hemodialysis patients, increased KYN/Trp ratio was found to be associated with atherosclerotic changes, such as decreased ankle-brachial pressure index and increased carotid artery intima-medial thickness (55). Hypercholesterolemia was shown to decrease enzyme activities of kidney 3-HAA 3,4-dioxygenase and liver TDO, thereby leading to a decreased formation of nicotinic acid (56). Application of kynurenines as prognostic parameters in CAD was also suggested (57, 58). The most frequently measured kynurenines are Trp, KYN, XA, AA and KYNA, and their ratios are also often determined. Various ratios of KP metabolites may provide indirect information about the activities of particular enzymes and/or show the relative activation of the three major branches of KP (25, 59, 60). KP metabolites have small molecular weight, and they have been found to be stable, measurable compounds (54). The within-person reproducibility for determination of KYN, KYNA, XA, 3-HK, AA, 3-HAA, and Kyn/Trp ratio in samples from chronic heart failure patients and control individuals was found to be good to fair (61), supporting the applicability of kynurenines as predictive biomarkers. The idea of using KP metabolites as biomarkers was first introduced more than 70 years ago, when elevated urine kynurenines were observed in patients diagnosed with cancer or cardiovascular disease (62, 63).

Reliable methods for determining Trp and its metabolites are essential for the utilization of kynurenines as biomarkers during the initiation and progression of CAD. However, a number of issues need to be considered in the development of analytical methods in order to obtain high robustness, selectiveness and sensitiveness. Hence, the physiological concentrations of endogenous Trp and its metabolites cover a wide range in various biological samples such as biofluids, cells or tissues. For instance, the physiological concentration of Trp was found in the µM range, while concentrations of KYN, KYNA and 3-HK were in the nM range in mice serum (64). The relatively low concentration of certain metabolites of KP and the presence of interfering compounds require the application of an effective sample preparation procedure as a key factor for assuring reliable measurement. The recovery and matrix effect of a given compound is determined by several physico-chemical factors related to itself and the applied condition of the method. For the analyte, pKa, pKb, pI, logP, stability, etc. are essential in the design of the sample preparation procedure (65). Regarding KP metabolites, these values cover a relatively wide spectrum, which makes it difficult to optimize a given sample preparation for all components without any compromise. Generally, the protein precipitation of biological samples is before the analysis using organic solvents and/or acids such as acetonitrile, methanol, trichloroacetic acid and formic acid (57, 66–69). The solid-phase extraction (SPE) procedures combined with extraction solvent evaporation provides an efficient, but labour-intensive and time-consuming approach for the enrichment of targeted kynurenines by removing interfering compounds (70–74). The automated online SPE methods are capable of decreasing the above-mentioned disadvantages of offline techniques (70). The complexity of the biological samples involves the presence of several isomers (isobars) with similar chemical structures. Their separation is also of great importance in achieving accurate qualitative and quantitative information about given biological matrices. Thereby, some analytical methods such as antibody-based procedures cannot be suitable for distinguishing isomers such as PA and nicotinic acid (69). The application of chromatographic separation techniques provides the opportunity to separate targeted KP metabolites from other endogenous isomers.

In the past, Trp metabolites were measured using UV spectrophotometry after separation by thin-layer chromatography followed by fluorometric identification and elution (75, 76); however, now these methods are considered outdated. Relevant analytical methods of the KP and their most important advantages and disadvantages are summarized in **Table 2**. The development of high-pressure liquid chromatography (HPLC) allowed monitoring KP metabolite levels in biological materials faster with higher sensitivity and accuracy than thin layer chromatography. HPLC combined with UV detection is a popular method for quantification of Trp, KYN, 3-HK, QA, PA and XA, since such instruments are widely available and the measurement is relatively easy (**Table 2**). However, this method has low selectivity due to interfering compounds in samples, chemical characteristics of measured molecules and different levels in biological samples (77). To improve sensitivity, fluorescence detectors coupled with HPLC are used for the analysis of kynurenines, which are frequently applied for the analysis of Trp and KYNA in blood, brain, heart or liver (87, 115) (**Table 2**). Another option for more accurate determination of kynurenines is the application of electrochemical detection combined with HPLC, a widely used method for quantification of Trp, KYN, 3-HAA, 3-HK, XA and AA (**Table 2**). The electrochemical detection is known for its high sensitivity; however, the main drawback of this approach is the lack of reproducibility caused by electrode clogging and loss in selectivity (115). The advent of ultra-high-performance liquid chromatography (UHPLC) provided enhanced chromatographic separation efficiencies on columns packed with sub-2 µm silica particles using UHPLC system with very low extra-column variance and high operating pressure (116). The main advantages of UHPLC techniques include: i) higher rate and throughput, i.e. separations can be achieved in a fraction of the time compared to that of HPLC; ii) better resolution and iii) sharper peaks and thus better lower limit of detection and quantification. The UHPLC method improved separation efficiency and analysis time of Trp metabolites and permitted to monitor multiple compounds in a single measurement, because of negligible co-elution of analytes (89, 117).

**Table 2:** Detection possibilities of Trp and kynurenine pathway (KP) metabolites and their most important advantages and disadvantages.

Detection method		Advantages	Disadvantages	Detectable Trp and KP metabolites	Origin of samples	Successful use of method for detection of Trp and KP metabolites
**HPLC** **(in general)**		Low-cost equipment	Relatively longer analysis time because of sample preparation (depending on the type of detection) High consumption of reagents High sample volume (depending on the type of detection) Complex workflows with intensively manual sample and information handling			
	**with UV absorbance detection**	Most suitable for clinical application in routine diagnostics	Lower sensitivity and selectivity, because of the detection	Trp, KYN, 3-HK, XA, PA, QA	Urine (human), sweat (human), serum (human), plasma (human), heart tissue (human), brain (rat), liver (rat), serum (rat)	(49, 77–85)
	**with fluorescence detection**	Higher sensitivity compared to UV detection	Suitable only for metabolites with autofluorescence Derivatization might be necessary	Trp, KYNA, 3-HAA, QA, AA	Serum (human), sweat (human), plasma (human), urine (human), brain (rat), liver (rat), placenta (rat), plasma (rat)	(49, 63, 77–79, 81, 83, 85–90)
	**with electrochemical detection**	One of the highest sensitivity among HPLC techniques	Low selectivity and reproducibility	Trp, KYN, 3-HK, 3-HAA, XA, AA	Brain tissue (mouse), serum (mouse), ileum (mouse), plasma (human), serum (human)	(49, 91–94)
	**with MS/MS detection**	High sensitivity and selectivity Applicability for multimetabolites analysis Relatively low reagent cost Requires minimum sample preparation and low sample volume (solid phase extraction) Low matrix effects and interferences High-throughput application Portability High separation efficiency	Careful multistep sample preparation (derivatization) Optimal standards needed for internal calibration Expensive equipment High costs of detection Low ionization response of Trp metabolites Higher signal to noise ratio compared to GC-MS (but with combination of appropriate detection system could be minimized)	Trp, KYN, 3-HK, 3-HAA, QA, XA, PA, AA, KYNA	Plasma (rat, human, mouse), cerebrospinal fluid (nonhuman primates), brain tissue (mouse), urine (human, mouse) serum (mouse) liver (mouse) intestinal content (mouse)	(24, 25, 36, 57, 61, 70, 72, 95–105)
**UHPLC-(HR)MS/MS**		High-throughput application Shorter analysis time Low sample volume Higher separation efficiency Less solvent consumption	Relatively new techniques Higher cost Not widely available Low ionization response of Trp Careful sample preparation	Trp, KYN, QA, KYNA, PA, XA, 3-HK, 3-HAA, AA	Cerebrospinal fluid (human), serum (human, mouse), plasma (human), urine (human, mouse), liver (mouse), intestinal content (mouse)	(65, 67–69, 73, 106–110)
**GC-MS**, **GC-MS/MS**		High sensitivity, mass resolution and accuracy High selectivity High Reproducibility Low sample volume Affordable, with relatively low running cost	Some of kynurenines are hardly detectable (KYN, 3-HK) Expensive equipments Requires additional sample preparation Lower mass accuracy	Trp, KYN, QA	Urine (human), plasma (human), brain (rat)	(61, 96, 111)
**ELISA**		Ready to use kits User friendly Small sample volume Widely available Relatively low cost Information about KP enzyme activity through the determination of KP metabolites	Not optimal for multi-metabolite analysis Not available for all kynurenines Lower specificity and sensitivity than LC-MS/MS Cross-reactivity issues	KYN, Trp, KYNA, QA, AA	Serum (human), plasma (human), urine	(25, 112)
**Fluorescent chemosensor**		High specificity Low cost Less-time consuming	Available only for KYN	KYN	Serum (human)	(113)
**Electrochemical immunosensor**		Suitable for lab-on-a-chip platform Low-cost Robustness Miniaturized and automatized detection	Not available for all kynurenines Only for blood samples	KYNA	Serum (human)	(114)

GC-MS, gas chromatography–mass spectrometry; GC-MS/MS, gas chromatography coupled with tandem mass spectrometry; HPLC, high-performance liquid chromatography; UHPLC-(HR)MS/MS, ultrahigh-performance liquid chromatography with (high resolution) tandem mass spectrometry; ELISA, enzyme-linked immunosorbent assay; Trp, tryptophan; KYN, kynurenine; 3-HK, 3-hydroxykynurenine, AA, anthranilic acid, 3-HAA, 3-hydroxyanthranilic acid; QA, quinolinic acid; KYNA, kynurenic acid; XA, xanthurenic acid; PA, picolinic acid.

The analysis of KP metabolites had been further advanced with the detection of the metabolites by mass spectrometry (MS), in particular, using instruments with high accuracy and mass resolution (HRMS). In order to obtain a highly selective and sensitive analysis of biological samples, MS is frequently hyphenated with chromatographic separation, for instance, gas chromatography (GC-MS) and liquid chromatography (LC-MS). Nowadays, the significance of GC-MS analysis of kynurenines has diminished with the advent of atmospheric ionization LC-MS methods. **Supplement Table 2** summarizes the main parameters of sample preparation procedures and LC-MS methods related to the analysis of Trp metabolites. LC-MS based metabolomics can be divided into two main strategies, i.e. non-targeted and targeted approaches. The primary aim of the non-targeted strategy is to obtain a comprehensive profile of the altered metabolites by providing mainly qualitative data, which are generally based on the accurate mass of metabolites obtained by hyphenated HRMS measurement. In comparison, the targeted approach focuses on quantitative or semi-quantitative information of a selected, limited number of metabolites commonly obtained by liquid chromatography coupled with tandem mass spectrometry (LC-MS/MS) (68, 118). The targeted LC-MS/MS analysis of kynurenines is based on monitoring retention times and precursor ions to product ions transitions, which are generally generated by positive ionization multiple reaction monitoring mode (MRM) (66, 70). The latest approach, using quadrupole-orbitrap hybrid high-resolution instruments in parallel reaction monitoring (PRM) mode, provides high specificity because the MS/MS data are acquired in high-resolution mode to separate the target ions from co-isolated background ions (65, 105). The PRM allows for parallel monitoring of a targeted precursor and all subsequent transition ions, while the MRM provides only one transition. However, the quadrupole-orbitrap hybrid high-resolution instrument has disadvantages, such as the higher cost and lower scan rate than triple quadrupole tandem mass spectrometers. Overall, the enhanced selectivity of the PRM methods provides better quantitative and qualitative results (119, 120). For data analysis, the obtained chromatographic peak area of the quantifier ion (most abundant fragment ion) provides quantitative information, while the peak area of qualifier ion (characteristic fragment ion) and the peak area ratio of the ions are used to confirm the presence of a given compound (103). An important consideration when comparing LC-MS methods is the lower limit of detection or quantification values obtained for the KP metabolites (**Supplement Table 2**). However, comparisons of these values are difficult due to the different methods of their determination, for instance, using matrix-free standard solution or metabolite free matrix (101, 103). A further aspect is the required amount of biological samples processed. With the development of LC-MS instruments and the analytical methods, the required amount of samples has been significantly reduced (67, 109). To demonstrate the possibility of simultaneous quantification of a large number of analytes with the LC-MS method, 30 different compounds of Trp metabolism were detected in human samples, including serotonin and indol pathways, as well as KP (115). To further broaden the possible measurable molecules and to handle the detection challenges, the HPLC-MS/MS method has been upgraded with an alternative sample preparation (ultrafiltration instead of protein precipitation) (101). Higher sensitivity was reached from lower sample volumes for adequate throughput for cost- and time-efficient routine sample analysis. The main limitation of LC was also highlighted, i.e., not all KP metabolites can be measured at sufficient sensitivity in all species and samples (101). Nevertheless, a single-run HPLC-MS/MS approach was successfully applied to the analysis of plasma samples from healthy and acute myocardial infarction patients (57). The methodology was further optimized by simplifying sample pretreatment and modifying reverse-phase separation to broaden the range of measurable Trp metabolites (104). On the other hand, LeFévre and his co-workers validated a UPLC-HRMS/MS method to measure Trp metabolites in mice urine, serum, intestinal contents and liver using Kinetex XB-C18 column (105). A high-throughput, sensitive and automated on-line solid-phase extraction–liquid chromatographic–tandem mass spectrometric (XLC–MS/MS) method applying positive electrospray ionization was also shown to enable accurate and precise measurement of Trp, KYN and 3-HK in plasma (70). The main advantages of XLC–MS/MS are relatively easy handling, portability, and reduction of cost per sample due to reduced sample preparation time, which can be even automatized to reduce analysis time and analytical variation caused by manual sample preparation and reuse of cartridges (70). Sensitivity and the range of measured Trp metabolites were further expanded using SPE and HPLC-MS/MS method for measuring Trp, KYN, KYNA, 3-HAA, AA, QA and PA in rat plasma, which allowed the analysis of a large number of samples in a single day (**Table 2**) (72).

The simultaneous detection of as many kynurenines as possible is one of the challenges in using Trp metabolites as potential predictive markers. However, high throughput capacity should be also handled during clinical practice. Hényková et al. set up an UHPLC technique connected to electrospray tandem mass spectrometry (UHPLC–ESI-MS/MS), wherein 18 Trp metabolites from the KP and serotonin pathway were measurable in human samples (**Table 2**). This method allowed accurate analysis of almost 100 samples in 24 h (106). Whiley and co-workers designed a targeted UHPLC-ESI-MS/MS in 96-well plate format for application in multiday, multiplate clinical and epidemiology population studies. A chromatographic cycle time of 7 min enabled the analysis of two 96-well plates in 24 h (109). The conception of using kynurenines as prognostic markers of a disease was strengthened by Schwieler et al. In their work, a novel, robust UHPLC-MS/MS method was used for quantification of multiple KP metabolites, including PA isomers, in the cerebrospinal fluid (69).

Validation of LC-MS methods by using relevant and optimal standards is rather challenging. Stable isotope-labeled kynurenines are the best candidates for internal standards, because of identical chemical properties to the target analyte. Using stable isotope-labelled internal standard, the instability, recovery, chromatographic behavior or ionization efficiency issues can be minimised in comparison to other internal standards. However, the main difficulties of this approach are high cost limiting its widespread use and the disadvantages of the optimal isotopically labelled internal standard. Specifically, the deuterium-labeled compounds may demonstrate unexpected behaviour, such as different retention times or recoveries (115); therefore, a possible solution can be the use of structural analogues.

To date, most of the HPLC-MS methods are time consuming, involve complex workflows with intensive handling of manual sample and information. As a result, laboratories need to spend time optimizing the workflow, which limits the overall throughput and delays time to reportable results. In addition, these methodologies require laborious method validation, and relatively costly and sophisticated equipment. Therefore, these methods currently are not widely adopted in clinical practice. Hopefully this situation will change soon due to continuous development of these techniques and it will not be a limitation for use in the near future.

Besides chromatographic techniques, enzyme-linked immunosorbent assay (ELISA) methods are also suitable for the specific and quantitative determination of several KP metabolites including KYN, Trp, KYNA, QA, AA and others (**Table 2**). The recognition of these metabolites by antibodies is linked to an enzymatic reaction leading to the formation of a coloured product that can be assayed by a plate reader for determining the concentration of the metabolite of interest in the sample. Standards are usually provided in the commercially available kits. These methods are suitable for the quantitative determination of KYN/Trp ratios (25) or KYNA concentrations (83) in different biological fluids. The main advantages are user-friendly application, small sample volume, relatively high throughput, and wide availability even in clinical laboratories. However, this technique is not available for all kynurenines. Usability of ELISA in prognostic investigations was strengthened by Bekki’s work, in which high level of serum KYN was correlated with poor prognosis of chronic hepatitis C virus infection and serum kynurenine was identified as an independent predictor for prognosis of patients (112). On the other hand, the determination of KYN/Trp can show the activity of IDO enzyme (25).

Recently, a new fluorescent chemosensor approach has been invented to detect KYN. KYN is incorporated as part of the fluorophore and functions through internal charge transfer induced bathochromic shift (113). This method was reported as a selective, convenient, less time-consuming and relatively cheap detection of KYN in human serum (**Table 2**). Furthermore, a novel electrochemical immunosensor with a multi-electrode platform was invented for detection of KYNA allowing a low-cost, robust, reliable, and non-invasive multi-analyte detection with miniaturised and automatized detection of KYNA in blood samples (**Table 2**) (114). These innovative methods, however, should be further developed to cover measurement of all KP metabolites.

In conclusion, various methods are available for the measurement of metabolites of KP, including KYN, KYNA, 3-HK or their ratio to Trp, that are potential prognostic markers of cardiovascular diseases. Choosing the most suitable methods for determination of kynurenines is really important to provide reliable and reproducible data for clinical decisions. The following aspects should be considered when deciding about the detection method to be used: the concentration of kynurenines and the suspected changes of metabolites levels due to the pathological conditions, advantages and disadvantages of different detection methods, such as sensitivity, selectivity, possible interference with other biological components, instrumental environment, price, and availability of infrastructure or expertise.

### 4.2 Atherosclerosis and KP

Atherosclerosis is a systemic disease that affects many vessel beds including aorta, carotid and coronary arteries as well. Carotid artery intima-media thickness, decreased ankle-brachial index and raised aortic stiffness are considered as clinical indicators of atherosclerosis, and these parameters correlate with the atherosclerosis of coronary arteries (121–123).

Main findings of our literature review focusing on KP metabolites as a marker of atherosclerosis are summarized in **Table 3**.

**Table 3:** Kynurenine pathway (KP) metabolites: markers of atherosclerosis.

Changes in Kynurenine Pathway metabolites’ blood level based on the endpoint of the investigation					Main Message	Investigated population	Number of participants	SexAge	Ref.
**KTR**	**KYN**	**TRP**	**OTHER**						
↑	↓	↓	3-HAA↑	in advanced atherosclerosis vs. control	KTR ratio (IDO activity) is positively and Trp is negatively associated and correlated with atherosclerosis KTR is positively associated with post-operative cardiac complications	patients with advanced atherosclerosis vs patients without atherosclerosis^1^	100 (22 control)	both sex 69-70 years	(73)
↑	↔	↓	3-HAA↔	represented high odds ratio for advanced atherosclerosis					
↑	↔	↓	3-HAA↑	correlated with decreased ankle-brachial index (ABI)					
↑	↑	↓	3-HAA↔	at baseline in post-operative complications vs. no complication					
↑	-	-	-	represented high odds ratio for post-operative complications					
↑	↔	↓	-	correlated with increased carotid artery intima-medial thickness (IMT) and maximal diameter of carotid plaques	elevated KTR ratio (IDO activity) may be related to advanced atherosclerosis in haemodialysis patients	patients undergoing regular haemodialysis	243	both sex 60 ± 10 years	(55)
↔	↔	↔	-	correlated with decreased ABI					
↑	-	-	-	associated with advanced atherosclerosis (increased IMT, plaque size, decreased ABI) vs lower KTR level					
↑^2^	↔	↓	-	in grade II-III atherosclerosis compared to normal group	KTR ratio (IDO activity) increases in atherosclerosis	patients with histologically verified atherosclerosis	51	both sex 42–91 years	(124)
↑	-	-	-	correlated with increased IMT	IDO activity is positively associated with atherosclerosis	older adults in Health 2000 Study	921	both sex 46–76 years	(125)
-	↑	-	3-HK↑ QA↑ KYNA↔ AA↔	correlated with increased IMT, and QA is independent predictor	disturbed kynurenine pathway may have a role in the atherosclerosis	patients with chronic kidney disease	106	both sex 55 ± 14 years	(49)
-	↑	-	QA↑ QA/KYN↑ KYNA↔	correlated with increased IMT and QA, QA/KYN are independent predictors	disturbed kynurenine pathway may have a role in the atherosclerosis	patients with end-stage renal disease	124	both sex 55 ± 14 years	(81)
↑	-	-	-	correlated with increased IMT in female subjects, but not in males	IDO enzyme is involved in immune regulation of early atherosclerosis in young female adults	young adults in Finn Study	986	both sex 24-39 years	(80)
-	-	-	KYNA ↑	correlated with increased aortic stiffness index and decreased aortic distensibility, aortic strain	disturbed KP may have a role in the pathogenesis of arterial stiffening	patients with persistent atrial fibrillation	100	43 females, 70 ± 8 years 57 males, 68 ± 7 years	(90)

^1^Patients underwent carotid endarterectomy, open infrainguinal revascularization or major leg amputation due to critical ischemia.

^2^Statistical significance is not indicated.

Studies are ordered alphabetically according to the first author. ↑ increase, ↔ not changed, ↓ decrease, **-** not examined; 3-HAA, 3- hydroxyanthranilic acid; 3-HK, 3-hydroxykynurenine; 5-HT, 5-hydroxytryptamine; AA, anthranilic acid; ABI, ankle-brachial index; IDO, indoleamine 2,3-dioxygenase; IMT, intima-medial thickness; KTR, kynurenine/tryptophan ratio; KYN, kynurenine; KYNA, kynurenic acid; QA, quinolinic acid; TRP, tryptophan.

Plasma KYN/Trp ratio was increased and Trp was decreased in patients with advanced atherosclerosis compared to patients without atherosclerosis (73, 124), and increased KYN/Trp ratio and low Trp represented high odds ratio for advanced atherosclerosis (73). Increased KYN/Trp ratio correlated with increased intima-media thickness (55, 80, 125) and decreased ankle-brachial index (73). Although Kato et al. found that ankle-brachial index was not correlated with either KYN/Trp ratio or Trp level, they showed that patients with high KYN/Trp ratio have advanced atherosclerosis (increased intima-media thickness, plaque size, decreased ankle-brachial index) compared to individuals with lower KYN/Trp ratio value (55). In addition, decreased Trp correlated with decreased ankle-brachial index (73) and increased intima-media thickness (55).

In patients with chronic kidney disease, two studies showed correlation between increased KYN, increased QA and increased intima-media thickness (49, 81), and one investigation failed to support KYN/Trp ratio-thickness correlation (55). Interestingly, Pawlak et al. have found that QA is an independent predictor for increased thickness in this population. KYN was not associated with decreased ankle-brachial index (55, 73).

Although 3-HAA was elevated in advanced atherosclerosis and correlated with decreased ankle-brachial index, it did not show increased odd ratio for advanced atherosclerosis (73). In atrial fibrillation increased KYNA was associated with raised aortic stiffness (90), but KYNA was not correlated with carotid intima-media thickness in chronic kidney disease (49, 81).

Taken together, disturbed KP is associated with atherosclerosis, and increased blood KYN/Trp ratio and decreased Trp seem to correlate with the severity of atherosclerosis, thereby KYN/Trp ratio and Trp may correlate with coronary atherosclerosis as well. Furthermore, determination of QA is promising in chronic kidney disease for prediction of the degree of atherosclerosis.

### 4.3 Coronary Artery Disease and KP

CAD is a group of distinct diseases like myocardial infarction, stable and unstable angina, sudden cardiac death, new onset of heart failure and so on. All CADs are characterized by atherosclerosis or atherosclerotic occlusion of the coronary arteries, but the severity and time-course of coronary atherosclerosis development result in a wide range of clinical manifestations that can be classified as either acute or chronic coronary syndromes (9). Emerging evidence suggests that low-grade inflammation contributes to the progression of atherosclerosis and CAD, also that the KP is essential for the modulation of these inflammatory responses (68, 99). The resulting increased KYN/Trp ratio is a measure of IFN-γ-mediated immune activation. In addition, IFN-γ activates the KP in monocytes (mainly through IDO1, KMO and QPRT upregulation) in a time-dependent manner and has been associated with risk of cardiovascular events (126, 127). Several metabolites in KP have been associated with different CADs including but not limited to acute coronary syndrome (99), chronic coronary syndrome (24) or post-cardiac arrest syndrome (98) (**Table 4**). It has been also demonstrated that some of the kynurenines are positively associated with CADs mortality and predicted increased risk of acute myocardial infarction in different patient populations (130). Studies examining circulating KP metabolites related to CAD as potential biomarkers are summarized in **Table 4**.

**Table 4:** Circulating kynurenine pathway (KP) metabolites related to coronary artery diseases (CAD): potential markers.

Changes in Kynurenine Pathway metabolites’ blood level based on the endpoint of the investigation					Main Message	Investigated population	Number of participants	Sex Age	Ref.
KTR	KYN	TRP	OTHER					
↑	↑	↓	3-HAA↔	at baseline who had later major adverse cardiac event (MACE)^1^	KTR may predict MACE in advanced atherosclerosis	patients with advanced atherosclerosis vs patients without atherosclerosis^2^	100 (22 control)	both sexes 69-70 years	(73)
↑	-	-	-	represented high odds ratio for MACE					
-	↑	-	-	associated with all-cause mortality	KYN was predictive for death and severity of heart failure, but KYN was no longer significant in multivariate model	chronic heart failure (44% had CAD)	114	both sexes 71 ± 12 years	(128)
-	↑	-	-	in NYHA III-IV vs. NYHA I-II correlates with severity of chronic heart failure (high NT-proBNP, low peak VO_2_, low LVEF, low GFR)					
-	↑	-	-	correlated with reduced LVEF and increased CAD severity	KYN was found to be correlated with chronic heart failure and CAD severity	chronic heart failure with implantable cardioverter-defibrillator (ICD) (71% had CAD)	156	both sexes 69 ± 11 years	(128)
-	↑	↔	KYNA↑ AA↔ 3-HK↑ XA↔ 3-HAA↔	at baseline associated with increased risk of acute coronary syndrome (ACS)^3^	KP can be involved in the early development of CAD and prediction of ACS	presumptively healthy elders without prior coronary events	2819	both sexes 71-74 years	(99)
↑	↑	-	-	significantly associated with in-hospital mortality	activation of the KP shows association with unfavourable clinical outcomes in cardiac arrest patients	cardiac arrest patients	270	both sexes 57-74 years	(110)
↑	↑	↓	-	significantly associated with poor neurological outcome^4^					
↔	↔	↔	3-HK↑ KYNA↔ XA↓ AA↔ 3-HAA↔ QA↔ 3-HK/XA↑	weakly associated with increased risk of all-cause mortality	increased 3-HK, 3-HK/XA and decreased XA had weak associations with increased mortality in CAD patients	CAD with preserved ejection fraction (i.e. without systolic heart failure)	807	both sexes 63 ± 10 years	(24)
↑	↑	↔	3-HK↑ KYNA↔ XA↔ AA↔ 3-HAA↔ QA↑ 3-HK/XA↑	in heart failure patients compared to controls with or without CAD^5^	heart failure itself can be associated with alterations of the KP, independent of CAD	heart failure (73.8% had CAD)	202	both sexes 63 ± 9 years	(24)
↑	↑	↔	3-HK↑ KYNA↔ XA↓ AA↔ 3-HAA↔ QA↑ 3-HK/XA↑	associated with increased risk of all-cause mortality	disturbed KP is associated with increased mortality in patients with heart failure				
↑	-	-	-	associated with risk of major coronary events (MCE)^6^	KTR can be a predictor of adverse prognosis, CVD and all-cause mortality in patients with stable angina pectoris and significant CAD	patients with stable angina pectoris and angiographically verified significant CAD	2380	both sexes 64 ± 10 years	(95)
↑	-	-	-	predicted CVD^7^ mortality					
↑	-	-	-	associated with all-cause mortality					
↑	-	-	-	in urine associated with increased CVD and all-cause mortality in dose-response manner	urine KTR is predictor of MCE, acute myocardial infarction (AMI), and mortality in stable CAD patients	patients with suspected stable CAD	3224	both sexes 62 ± 11 years	(96)
↑	-	-	-	in urine associated with increased incidence of MCE and AMI in dose-response manner					
↑	-	-	KYNA↑ 3-HK↑ AA↑ 3-HAA↑ XA↔	associated with incidence of AMI	KTR and disturbed KP pathway increases the risk of AMI in CAD patients	suspected stable angina pectoris	4122	both sexes 55-70 years	(100)
↑	↔	↓	KYNA↔ 3-HAA↔	in cardiac arrest patients compared to healthy controls	KP is associated with the severity of post-cardiac arrest shock, early death, and poor long-term outcome all KP metabolites were independent predictors of early death, while KYNA and 3-HAA were independent predictors of poor 12-month neurological outcome as well	cardiac arrest patients with both shockable and nonshockable initial rhythms	245 (10 control)	both sexes 53-72 years	(98)
↑	↑	↔	KYNA↔ 3-HAA↑	in cardiac arrest patients with nonshockable initial rhythm compared to patients with initial shockable rhythm					
↑	↑	↔	KYNA↑ 3-HAA↑	in patients with lower blood pressure and lower bicarbonate levels during the first 24 hrs after return of circulation					
↑	↑	↔	KYNA↑ 3-HAA↑	correlated with intensive care unit death, 12-month death and poor neurological outcome					
↓	↓	↔	KYNA↔ XA↔ 3-HAA↔ QA↔ PA↔ *FA*	in CAD patients vs non-CAD patients (post-mortem)	post-mortem KTR and disturbed KP pathway may predict severe CAD	individuals died from sudden unexpected death with severe CAD occlusion more than 75% of the cut surface	31	male 21-86 years	(84)
↓	↓	↓	KYNA↓ 3-HAA↓ QA↓ PA↑ *FA*	associated with severity of CAD occlusion^8^					
↑	-	↓	KYNA↓ 5-HT↔	in hypothermia compared to baseline	IDO becomes activated under hypothermia, and may contribute to increased susceptibility to infection/sepsis under lower body temperatures	post cardiac arrest patients treated with target temperature management	20	both sexes 54-74 years	(107)
↑(NS)	-	↓(NS)	KYNA↑ 5-HT↔	in patients with poor neurological outcome compared to the ones with favourable outcome					
↑	-	-	-	at baseline associated with increased risk of ACS^9^	KTR level predicts ACS	older adults without previous CAD	2743	both sexes 71-74 years	(129)
↑	↑	↓	KYNA↔ XA↔ 5-HT↓	in AMI compared to healthy controls free from CVD	KP metabolite might be biomarkers for monitoring of AMI progression	hospitalized patients diagnosed with acute myocardial infarction	9 (18 controls)	both sexes N.A.	(57)
-	-	↑	KYNA↓ XA↓	in urine samples of ACS patients compared to healthy controls	as a part of wide urinary metabolomics KP metabolites may serve as biomarkers in ACS diagnosis	ACS patients	36 (30 controls)	both sexes 59 ± 8 years	(68)
↑	↔	↓	-	in CAD vs. healthy controls	KTR may be involved in the development of CAD	CAD verified by coronary angiography	35 (35 controls)	both sexes 61 ± 10 years	(79)
↑(NS)	↔	↔	-	among 1-vessel, 2- or 3-vessel CAD and restenosis groups					
↑	↑	↔	-	in significant CAD vs non-significant CAD, and it was predictive for significant CAD	KTR may predict CAD severity	patients with suspected CAD	305	both sexes 64 ± 10 years	(36)
↑	↑	-	-	correlated with the CAD severity					
↓	↓	-	-	in single-vessel CAD/non-significant CAD patients at baseline who had MCEs^10^ later	disturbed KP pathway might be associated with poor outcome in CAD patients				
↔	↔	-	-	in double- and triple-vessel CAD patients at baseline who had MCEs later					
↓(NS)	↓(NS)	-	-	tendentiously at baseline in patients who died later					
↑	↑	↓/↔^11^	-	associated with increased all-cause mortality	KYN was associated with all-cause mortality in two independent prospective cohorts of patients with ICD, as well as with ventricular arrhythmia-induced ICD schocks	ischemic or non-ischemic systolic heart failure with implantable cardioverter-defibrillator (ICD)	402 (PROSE-ICD) 240 (GRADE)^12^	both sexes 18-80 years	(67)
↔	↑/↔^10^	↔	-	associated with increased ventricular arrhythmia-induced ICD shocks					
↑	↑	↓	KYNA↔ 3-HK↑ AA↑ 3-HAA↔ XA↓	at baseline associated with increased CVD mortality^13^	KTR and disturbed KP pathway may predict CVD mortality	individuals with or without any kind of diseases (e.g. CVD, diabetes, etc.)	7015	both sexes 46-49 years 70-74 years	(102)

Studies are ordered alphabetically according to the first author. ↑ increase, ↔ not changed, ↓ decrease, **-** not examined; 3-HAA, 3- hydroxyanthranilic acid; 3-HK, 3-hydroxykynurenine; 5-HT, 5-hydroxytryptamine; AA, anthranilic acid; ACS, acute coronary syndrome; AMI, acute myocardial infarction; BNP, brain natriuretic peptide; CVD, cardiovascular disease; FA, further ratios are available; GFR, glomerular filtration rate; ICD, implantable cardioverter-defibrillator; IDO, indoleamine 2,3-dioxygenase; IMT, intima-medial thickness; KTR, kynurenine/tryptophan ratio; KYN, kynurenine; KYNA, kynurenic acid; LVEF, left ventricular ejection fraction; MACE, major adverse cardiac event; MCE, major coronary events; NS, non-significant; NYHA, New York Heart Association functional classification; PA, picolinic acid; QA, quinolinic acid; TRP, tryptophan; XA, xanthurenic acid.

^1^all-cause death, stroke, myocardial infarction, coronary revascularization during the follow-up period.

^2^patients underwent carotid endarterectomy, open infrainguinal revascularization or major leg amputation due to critical ischemia.

^3^unstable angina pectoris, AMI, sudden death in crude analysis (adjusted for gender); only KYN and HK significant when adjusted for gender, hypercholesterolemia, kidney function (eGFR), smoking, BMI, hypertension, and diabetes.

^4^after adjusting for age, gender and comorbidities, only ↑ KTR remains significantly associated with poor neurological outcome.

^5^adjusted for diabetes, eGFR, pyridoxal 5’phosphate, C-reactive protein and Trp (not Trp in KTR model).

^6^fatal and non-fatal AMI, sudden cardiac death, sudden death.

^7^International Classification of Diseases (ICD)-10 codes I00-I99 or R96.

^8^only PA is significant in trend correlation.

^9^unstable angina, non-fatal or fatal AMI or sudden death.

^10^death, myocardial infarction, and/or recurrent cardiac chest pain.

^11^results were different in PROSE-ICD study/in GRADE study.

^12^models were adjusted for age, sex, race, enrolment center, smoking status, BMI, LVEF, NYHA class, atrial fibrillation, diabetes, hypertension, and CKD (adjustment for kidney disease was only done in PROSE-ICD as the information was not available in GRADE).

^13^associciation was non-significant in participants without self-reported cancer, CVD (myocardial infarction, angina, and stroke), or diabetes.

The importance of KP in influencing cardiovascular disease mortality (analysis of cause specific mortality) was investigated in Hordaland Health Study (99, 102). In this cohort study, the plasma CRP indicating chronic inflammation was positively correlated with KYN, 3-HK, and 3-HAA and negatively correlated with XA and tryptophan in age- and sex adjusted analyses (102). KYN, AA, and HK were positively associated with risk of all-cause mortality; however, Trp and XA were inversely associated with mortality risk (102). There were no linear associations between KYNA, 3-HAA and risk of all-cause mortality. Increased baseline KYN/Trp, KYN, 3-HK, AA and decreased Trp, XA were related to later cardiovascular death (**Table 4**) (102). KYNA and 3-HAA showed no association. IFN-γ-mediated inflammation and activation of KP seem to have a stronger relationship with cardiovascular mortality than with mortality due to cancer or other causes (102). Interestingly, the elevated baseline KYN/Trp ratio predicted higher risk for acute coronary event (like unstable angina, non-fatal or fatal acute myocardial infarction or sudden death) in older patients without prior coronary disease (129) (**Table 4**), therefore this study showed that the KYN/Trp ratio may predict future coronary events years ahead of the acute episode, among community-dwelling older adults without prior coronary heart disease.

Wirleitner and co-workers investigated the concentrations of KYN, free Trp, and neopterin, as well as the KYN/Trp ratio in blood samples of angiographically verified CAD patients collected before transluminal coronary angioplasty (79). According to their findings, KYN/Trp ratio was increased and Trp was decreased in CAD compared to controls (**Table 4**), and Trp degradation correlates with the levels of neopterin, the formation of which is stimulated by IFN-γ, suggesting that the lowering of Trp concentration is caused by the IFN-γ-induced stimulation of IDO and subsequent activation of KP. Therefore, reduced availability of both Trp and Trp-derived serotonin, as well as the production of toxic compounds through the ‘bad’ arm of the KP, such as QA may contribute to the development of neuropsychiatric disorders in CAD patients (79).

It is worth to mention that postmortem analysis of blood Trp metabolites as possible biomarkers for CAD might contribute to the investigation of sudden unexpected deaths (84). Several Trp metabolites were analyzed to help the differentiation between non-CAD and CAD pathologies after sudden unexpected deaths. Decreased KYN/Trp, KYN, PA/KYNA and increased PA/KYN, PA/3-HAA, were observed in CAD-caused deaths (**Table 4**). No significant differences have been identified in Trp, KYNA, 3-HAA, XA, QA, PA levels between groups. Authors have proposed that PA/KYNA and PA/3-HAA may be suitable markers for classifying non-CAD out of the CAD in sudden unexpected deaths (84).

Besides kynurenine metabolites, the synthetizing enzymes might be also used as prognostic parameters. IDO becomes up-regulated or upregulated in response to various infectious and inflammatory stimuli. The importance of IDO enzymes in prognosis of cardiovascular diseases was supported by Li’s work (131), where a Mendelian Randomization was used to obtain unconfounded estimates of the association of IDO1 with ischemic heart disease, ischemic stroke and their risk factors. The IDO1 protein showed inverse association with ischemic heart disease, with its risk factor, diabetes mellitus type 2, but it was not clearly associated with systolic or diastolic blood pressure. They concluded that the life-long increased plasma IDO1 was inversely associated with risk of developing ischemic heart disease.

Correlation between severity of CAD and KP metabolites were also investigated (**Table 4**). In patients with suspected CAD, increased KYN/Trp ratio and KYN were predictive for significant CAD and both changes correlated with CAD severity (36). Post-mortem analysis have revealed that decreased KYN/Trp ratio, KYN, Trp, KYNA, 3-HAA, QA, 3-HAA/Trp, QA/PA, QA/Trp and increased PA, Trp/KYNA, PA/3-HAA, PA/KYN, PA/Trp, PA/KYNA correlated with severity of CAD occlusion (84). In chronic heart failure with implantable cardioverter-defibrillator KYN was found to be correlated with CAD severity (128).

The alteration of KP metabolites is also associated with the cardiovascular risk factors, review in detail (21). For instance Eussen et al. showed that the plasma concentrations of kynurenines were generally higher in participants with hypertension, overweight and the KP was found to be dysregulated in obese individuals (99). A significant correlation between IDO and BMI, waist circumference and waist-to-hip ratio were observed in Pertovaara’s work (80). Positive correlation of increased IDO1 activity and KYN with incidence of CAD and low-grade inflammation, obesity, dyslipidemia, insulin resistance, diabetes and metabolic syndrome were also demonstrated (80, 100, 132). Diabetes mellitus is one of the major risks for CAD and it can be described as chronic low-grade inflammation, in which the macrophages actively contribute to the development of atherosclerotic plaques. Moreover, the proinflammatory cytokine, IFN-γ can modulate the activity of rate-limiting enzyme of KP, the IDO1, therefore the dysregulation of the KP might be involved in the pathogenesis of CAD and/or its risk factors, including diabetes mellitus (133). Risk for CAD is also influenced by the sex as well (134). It was identified that the sex has influence on the amount of KP metabolites, inducing different blood levels of kynurenines and Trp among males and females, which is further complicating the relation of kynurenines to CAD, however, the correlation between the KP changes and CAD remained identical (84). Substantial data suggest that the sex difference in the activity of KP is caused by mainly hormonal factors (135). In *in vivo* experiments, it has been revealed that the combined effect of estrogen and corticosterone upregulates the activity of the KP in rats. The modulatory role of female sex hormones on KP was supported by human studies, which has revealed that the KYN/Trp rises during pregnancy, and in oral contraceptive users as well. On the other hand, it has been confirmed that administration of androgens to both male and female subjects reduces the excretion of Trp metabolites *via* urine (135). It can be mentioned that people with anemia had a decrease in blood Trp level, which is positively correlated with a drop of hemoglobin (126).

#### 4.3.1 Acute Coronary Syndrome and KP

ACS is a severe type of CAD, which is usually associated with atherosclerotic plaque rupture and thrombus formation leading to acute myocardial infarction (AMI), unstable angina, acute heart failure, arrhythmias or even sudden cardiac death.

In the Hordaland Health Study, more than presumptively 2500 healthy elders without prior coronary events were involved to study the plasma concentration of KP metabolites and its potential linkage to acute coronary event endpoints (99, 129). During the investigation, significant positive associations of increased baseline concentrations of KYN/Trp, KYN, KYNA, and 3-HK with risk of ACS were described (**Table 4**) (99, 129). Among the kynurenines, KYN and 3-HK showed the strongest relations with markers of cellular immune activation and were associated with increased risk of acute coronary events in community-dwelling elderly without a known history of CAD. Circulating Trp, AA, XA and 3-HAA were not associated with increased risk of ACS (99).

It was also demonstrated, in patients with stable angina pectoris, that systemic markers of IFN-γ activity, plasma neopterin, and increased plasma KYN/Trp ratio provide similar risk estimates for ACS-related major coronary event, like AMI or sudden cardiac death (**Table 4**) (95). Therefore, the elevated levels of neopterin and KYN/Trp ratio possibly identify subjects with vulnerable lesions despite a clinically stable condition (95).

Besides blood specimens, urine samples can be used as non-invasively collected sources of biomarkers during diagnosis of cardiovascular diseases. Wand et al. applied urine metabolomics to investigate potential biomarkers and metabolic profiles for the prediction and diagnosis of ACS (68). In this study, Trp concentration was significantly increased in urine samples with parallelly decreased KYNA and XA levels compared to healthy controls (**Table 4**) (68). Pedersen et al. published that in suspected CAD increased urine KYN/Trp ratio was associated with increased incidence of ACS-related major coronary event, like AMI or sudden cardiac death (**Table 4**) (96). Another interesting finding was that KYN/Trp ratio seemed to be relatively stable over time in urine samples, which raises the chance of its clinical application as a biomarker in the future.

In advanced atherosclerosis, increased KYN/Trp ratio, KYN and decreased Trp were associated with increased major adverse cardiac event (including AMI and coronary revascularization, all-cause death, stroke) in the follow-up period (**Table 4**) (73). Furthermore, solely KYN/Trp ratio represented high odds ratio for later major adverse cardiac event (73).

Wongpraparut et al. have found that decreased baseline KYN/Trp ratio and KYN in single-vessel or insignificant CAD was associated with major cardiac events (like death, myocardial infarction, and/or recurrent cardiac chest pain) in the 1-year follow-up period (**Table 4**) (36). Regarding these levels, there were no association with cardiac events in double- or triple-vessel CAD patients.

##### 4.3.1.1 AMI and KP

AMI means myocardial cell death, an injury caused by sudden coronary occlusion related to ischemia usually as a consequence of CAD. Based on our research, few studies focused specifically on the relation between AMI and KP (**Table 4**). According to Tong’s work, the plasma concentration of Trp, kynurenines, 5-hydroxytryptamine (5-HT), as well as the concentration ratio of KYN/Trp and Trp/5-HT might serve as biomarkers to monitor the initiation and progression of AMI and evaluate the outcomes of therapeutic agents (57). In AMI compared to healthy controls, increased level of KYN/Trp, KYN and decreased Trp, 5-HT was measured without any changes in KYNA or XA (57).

Pederson et al. have found that increased level of KYN/Trp in the urine (96) and the plasma (100) is associated with the risk of AMI in suspected CAD patients. They have also demonstrated that increased circulatory KYNA, 3-HK, AA, 3-HAA were associated with incidence of AMI in CAD patients (100) and association was stronger with adverse prognosis among patients with impaired glucose homeostasis. Furthermore, the addition of AA improved goodness of fit for the multivariable model and AA provided significant net reclassification improvements (100).

These results were strengthened by Lewis et al., where the metabolomic platform of patients was utilized to discover blood markers with potential to detect the presence of myocardial injury. They found that AA becomes elevated for sustained periods after 2 hours of planned AMI (i.e. alcohol septal ablation treatment for hypertrophic obstructive cardiomyopathy). This finding suggests both the alteration of Trp metabolism during cardiac ischemia and its potential role in the response to ischemia (136).

##### 4.3.1.2 Cardiac Arrest and Post-Cardiac Arrest Syndrome and KP

Sudden cardiac death, also known as sudden cardiac arrest, occurs when the heart abruptly stops beating. CAD is the most common cause of cardiac arrest (137) which consequently leads to circulatory shock, i.e. an imbalance between oxygen demand and supply caused by inadequate blood flow, resulting in cell dysfunction and cell damage. Despite initial successful cardiopulmonary resuscitation, the morbidity and mortality following cardiac arrest remain high. The pathological state called post-cardiac arrest syndrome is characterized by cardiac dysfunction with circulatory shock and systemic inflammation.

The early activation of the KP after successful resuscitation was recently demonstrated (**Table 4**) (98). It was shown that Trp was significantly lower and the ratio of KYN to Trp was significantly higher in all resuscitated patients compared to healthy volunteers, and significantly higher levels of KP metabolites were observed in patients who died compared to those who survived. Elevated KYNA and 3-HAA levels were associated with 12-month poor neurological outcome. KP metabolites KYN, KYNA, and 3-HAA were markedly increased in the instance of poor outcome, supporting the specific prognostic role of KP. “Although KYNA generation might represent a protective adaptive response to overcome the neurotoxic effects resulting from 3-HAA, the ratio of KYNA to 3-HAA was not significantly different in patients who survived compared to those who died, and did not correlate with outcomes neither” (98). These findings were also observed in rats and pigs, where the increased plasma levels of KYN, KYNA and 3-HAA occurred during the initial hours following resuscitation and persisted up to 3–5 days following cardiac arrest and KP activation showed an equivalent time course in rats, pigs, and humans, and was significantly related to the severity of post-resuscitation myocardial dysfunction, functional outcome and survival (97). KYNA seems to be neuroprotective against ischemic brain damage caused by global or focal cerebral hypoperfusion (138–141), but probably the effect of endogenously raised KYNA is blunted by several factors (e.g. comorbidities, age, gender, etc.) or the production rate of KYNA was not enough to overcome the neurotoxic effects, which would explain the lack of correlation between increased level of KYNA and good prognosis in studies conducted by Ristango et al.

Another prospective cohort study investigating the possible relationships between the activation of KP and the mortality of cardiac arrest patients revealed that both increased KYN concentration and KYN/Trp ratio is significantly associated with in-hospital mortality (**Table 4**) (110). In the same study, the connections between KP and neurological outcome were examined too as a secondary endpoint. Lower Trp and higher KYN, with subsequent higher KYN/Trp ratio were significantly associated with poor neurological outcome at hospital discharge in cardiac arrest patients, which can be explained by the influx of KYN and neurotoxic compounds of the KP during cerebral hypoxia occurring upon cardiac arrest (110).

A study was conducted by Schefold et al., where they examined the effect of controlled body temperature after cardiac arrest on the serum level of Trp, KYN/Trp ratio and KYNA (107). Cardiac arrest causes global hypoperfusion (i.e. ischemia) to the body (including brain as well) and a subsequent cell dysfunction and damage, accompanied by global inflammatory response. Controlled hypothermia (decreased body temperature to 32–34°C) is used in clinical practice to ease systemic inflammation and hypoperfusion associated dysfunction and damage. They showed that hypothermia results in increased KYN/Trp ratio, decreased Trp and KYNA (**Table 4**). They also determined the neurological outcome according to Pittsburgh Cerebral Performance Category (CPC), which is a scale on 1 to 5. 1 is e.g. good cerebral performance: conscious, alert, able to work, might have mild neurological or physiological deficit contrast to 5 which is brain death (142). Interestingly, they found higher KYNA, non-significantly higher KYN/Trp ratio and lower Trp level in patients with unfavorable neurological outcome (CPC 3-5) vs. favorable outcome (CPC 1-2) (107).

In one cohort of systolic heart failure with undefined CAD status, increased blood KYN was associated with increased ventricular arrhythmia-induced implantable cardioverter-defibrillator shocks as an indicator of potential cardiac arrest caused by arrhythmia (**Table 4**) (67). Nevertheless, they have not found such a significant association in another cohort study with the same outcome (67).

#### 4.3.2 Chronic Coronary Syndrome and KP

Stable angina and ischemic chronic heart failure are common manifestations of chronic coronary syndrome of CAD.

It is known that metabolites of the KP mediate immunomodulation, oxidant defense and apoptosis, for detailed information please see review articles: (143–145). These mechanisms are involved in the development of heart failure (146, 147); therefore, the abnormalities of KP may influence the development and progression of heart failure (128). This phenomenon was strengthened by Lund et al., where the adjusted KYN, 3-HK, QA and derived ratios KYN/Trp and 3-HK/XA were higher in heart failure patients compared to control subjects independently from CAD status (**Table 4**), and these elevated levels associated with higher all-cause mortality in heart failure. Interestingly, increase in XA was consistently related to lower mortality in investigated heart failure and control groups (24). Increasing evidence suggest the predictive value of kynurenines as biomarkers in chronic heart failure (61, 67, 128, 148). It has been demonstrated that KYN increased with severity of chronic heart failure and performed better than NT-proBNP for predicting mortality and reflect exercise capacity. In a logistic regression analysis, KYN proved to be the only parameter among a number of chronic heart failure, inflammatory and oxidative stress markers that showed predictive value for death and reflect exercise capacity (**Table 4**) (128). In this univariate analysis KYN predicted all-cause mortality compared to NT-proBNP that was not predictive for death (128). Furthermore, ROC curve for KYN tended to be higher than for NT-proBNP. KYN and NT-proBNP similarly predicted severely compromised left ventricular function, but they were not compared in assessing exercise capacity. Based on death prediction analysis, authors proposed KYN as a better marker (128). The increased KYN concentration was also measured and associated with all-cause mortality and appropriate shock in two independent prospective cohorts of heart failure patients undergoing implantable cardioverter-defibrillator implantation for primary prevention of sudden cardiac death (**Table 4**) (67).

## 5 Conclusion

Although several detection methods are available for the measurement of KP metabolites, there is still no consensus about standardized assays suitable for widespread and routine use in clinical laboratory diagnostics. Moreover, determination of population-wide normal concentration ranges for individual metabolites of KP are urgently needed. Simultaneous determination of multiple components of KP may be straightforward in assessing the link between KP and various manifestations of CAD; however, large scale clinical studies are required to provide strong evidences.

Based on reviewing the literature, we can conclude that disturbed KP is associated with atherosclerosis, and more specifically increased blood KYN/Trp ratio and decreased Trp seem to correlate with the severity of atherosclerosis, thereby KYN/Trp ratio and Trp may correlate with coronary atherosclerosis as well. Furthermore, determination of QA is promising in chronic kidney diseases for prediction of the degree of atherosclerosis. Measurement of KYN and Trp, and calculation of their ratio can help to predict the degree of atherosclerosis and to follow-up the efficacy of anti-atherosclerotic treatment; nevertheless, large-scale clinical studies are still needed to determine the normal range of KYN/Trp ratio in healthy individuals and the cut-off value for atherosclerosis prediction. Change in KYN/Trp ratio itself is not specific for atherosclerosis, so it is not really suitable for diagnosis. Only few studies examined the possible predictive role of other KP metabolites with inconclusive findings, so further investigations are needed to elucidate this role in atherosclerosis.

Disturbed KP - especially increased KYN/Trp ratio and KYN – have been also found to be associated with unfavourable overall outcome in healthy individuals, patients with CAD, heart failure and cardiac arrest. However, due to the fact that a limited number of studies have been performed on a highly heterogeneous patient population applying various detection methods and targeting different KP metabolites and clinical endpoints, it is not surprising that drawing conclusions on any clear disease- or outcome-specific difference in KP changes which allows exact diagnosis of suspected disease or predicting specific endpoints is rather challenging. Nevertheless, measuring and adding KYN/Trp ratio and/or KYN to cardiovascular risk assessment and stratification seems to improve the accuracy of prediction of important outcomes like incidence of CAD, cardiovascular diseases and all-cause mortality. Follow-up of KP metabolites may be suitable for monitoring of severity and progression of CAD and heart failure, and also the efficacy of therapy. Measurement of 3-HK seems to be promising in prediction, and AA may improve the prediction of AMI in suspected CAD patients. Comprehensive analysis of whole KP metabolome was conducted in only few studies; therefore whole metabolome approach is recommended for future investigations to better understand the possible roles of different KYN branches in particular manifestations of CAD.

## Author Contributions

RG, MP, and TC conceptualized the work. All authors reviewed the literature, wrote and prepared the manuscript. RG, DH, VD, RB, and MP prepared the tables. VD prepared the figure. TC and MP critically revised and edited the manuscript. All authors have read and agreed to the submitted version of the manuscript.

## Funding

The work and publication were supported by the projects GINOP-2.3.2-15-2016-00034, EFOP-3.6.2-16-2017-00006 (LIVE LONGER), TKP2021-EGA-32, OTKA-NKFIH (FK138992) and by the Ministry of Human Capacities (20391- 3/2018/FEKUSTRAT). RG, DH and VD were supported by the New National Excellence Program of the Ministry of Human Capacities (ÚNKP-20-4-SZTE-150, ÚNKP-20-2-SZTE-64, ÚNKP-19-3-SZTE-47).

## Conflict of Interest

The authors declare that the research was conducted in the absence of any commercial or financial relationships that could be construed as a potential conflict of interest.

## Publisher’s Note

All claims expressed in this article are solely those of the authors and do not necessarily represent those of their affiliated organizations, or those of the publisher, the editors and the reviewers. Any product that may be evaluated in this article, or claim that may be made by its manufacturer, is not guaranteed or endorsed by the publisher.

## Abbreviations

3-HAA, 3-hydroxyanthranilic acid; 3-HK, 3-hydroxykynurenine; AA, anthranilic acid; ACS, acute coronary syndrome; AMI, acute myocardial infarction; CAD, coronary artery disease; ELISA, enzyme-linked immunosorbent assay; GC, gas chromatography; HPLC, high-performance liquid chromatography; HRMS, high accuracy and mass resolution; IDO, indoleamine 2,3-dioxygenase; KAT, kynurenine aminotransferase; KMO, kynurenine monooxygenase; KP, kynurenine pathway; KYN, kynurenine; KYNA, kynurenic acid; LC, liquid chromatography; MRM, multiple reaction monitoring mode; MS, mass spectrometry; MS/MS, tandem mass spectrometry; PA, picolinic acid; QA, quinolinic acid; QPRT, quinolinate phosphoribosyltransferase; PRM, parallel reaction monitoring; SPE, solid-phase extraction; TDO, tryptophan-2,3-dioxygenase; Trp, tryptophan; UHPLC–ESI-MS/MS, ultrahigh performance liquid chromatography–electrospray ionization tandem mass spectrometry; XA, xanthurenic acid; XLC, automated on-line solid phase extraction method coupled to high performance liquid chromatography.
