# Supplementary material for: Kynurenine Pathway Metabolites as Potential Clinical Biomarkers in Coronary Artery Disease
Source: Front Immunol. 2022 Feb 8;12:768560. doi: 10.3389/fimmu.2021.768560 (PMC8861075; doi:10.3389/fimmu.2021.768560)
Supplement: Supplementary file 1 [file Table_1.docx]

**Supplement Table 1. Cell type-specific RNA expression of enzymes involved in KP.** Table shows expression intensity of genes in human single cells from healthy tissue samples (except blood). Normalized transcripts per million values (nTPM) < 1 is considered as not detected (ND). nTPM < 10: +, nTPM >10: ++, nTPM > 100: +++, nTPM > 1000: ++++. Table is based on single cell transcriptomics datasets from www.proteinatlas.org ([Karlsson et al., 2021](#_ENREF_1)). AFMID: kynurenine formamidase; HAO: 3-hydroxyanthranilate 3,4-dioxygenase;IDO: indoleamine 2,3-dioxygenase, KAT: kynurenine aminotransferase; KMO: kynurenine 3-monooxygenase; KYNU: kynureninase, TDO: tryptophan 2,3-dioxygenase. No available data for anthranilate 3-monooxygenase.

| **Enzyme** | **Cardiomyocyte** | **Endothelial cell** | **Fibroblast** | **Smooth Muscle Cell** | **Macrophage** | **Monocyte** | **Dendritic cell** |
| --- | --- | --- | --- | --- | --- | --- | --- |
| IDO1 | ND | + | ND | + | ++ | ++++ | ND |
| IDO2 | ND | ND | ND | ND | ND | ++ | ND |
| TDO | ND | + | + | ND | + | ND | ND |
| AFMID | ++ | ++ | ++ | ++ | ++ | + | + |
| KYNU | + | + | + | + | +++ | +++ | + |
| KMO | ND | ND | ND | + | ++ | + | + |
| KAT1 | ND | ND | ND | + | ND | ND | ND |
| KAT2 | + | ND | + | + | ND | ND | ND |
| KAT3 | ND | + | ++ | + | + | ++ | ++ |
| KAT4 | +++ | ++ | ++ | ++ | ++ | ++ | ++ |
| HAO | ND | + | ++ | ++ | + | + | + |

Karlsson, M., Zhang, C., Mear, L., Zhong, W., Digre, A., Katona, B., Sjostedt, E., Butler, L., Odeberg, J., Dusart, P., Edfors, F., Oksvold, P., Von Feilitzen, K., Zwahlen, M., Arif, M., Altay, O., Li, X., Ozcan, M., Mardinoglu, A., Fagerberg, L., Mulder, J., Luo, Y., Ponten, F., Uhlen, M., and Lindskog, C. (2021). A single-cell type transcriptomics map of human tissues. *Sci Adv* 7.
