# Supplementary material for: Kynurenine Pathway Metabolites as Potential Clinical Biomarkers in Coronary Artery Disease
Source: Front Immunol. 2022 Feb 8;12:768560. doi: 10.3389/fimmu.2021.768560 (PMC8861075; doi:10.3389/fimmu.2021.768560)
Supplement: Supplementary file 2 [file Table_2.docx]

**Supplement Table 2.** **Main parameters of LC-MS analytical methods for determination of Trp and its metabolites. Abbreviation for methodology:** SPE: Solid-phase extraction; ESI: electrospray ionization mode; TFA: trifluoroacetic acid; ACN: acetonitrile; FA: formic acid; TCA: trichloroacetic acid; HFBA: heptafluorobutyric acid; PBS: phosphate-buffered saline; PRS: propyl sulphonic acid based strong cation exchange; LLOD: lower limit of detection; LLOQ: lower limit of quantification; MeOH: methanol; LLE: liquid Liquid Extraction; GC: gas chromatography; LC: liquid chromatography; UHPLC: ultrahigh performance liquid chromatography; XLC: automated on-line solid phase extraction method coupled to high performance liquid chromatography; MS/MS: tandem mass spectrometry; HRMS/MS: high resolution tandem mass spectrometry; ESI MRM: electrospray ionization multiple-reaction monitoring; ESI PRM: electrospray ionization parallel reaction monitoring; Abbreviations for kynurenine pathway: 3-HAA: 3-hydroxyanthranilic acid; 3-HK: 3-hydroxy-kynurenine; AA: anthranilic acid; KYN: L-kynurenine; KYNA: kynurenic acid; QA: quinolinic acid; XA: xanthurenic acid; PA: picolinic acid; Trp: tryptophan

| **LC-MS method** | **Type of samples** | **Main parameters of the sample preparation procedure** | **Main chromatographic conditions** | **Type of MS detection** | **LLOD or LLOQ of TRP and its metabolites** | **Reference** |
| --- | --- | --- | --- | --- | --- | --- |
| HPLC-MS/MS | mouse plasma, cerebro-spinal fluid, brain | **Plasma:** 10 µL plasma, 10 µL internal standard, 150 µL ice-cold MeOH, centrifugation, evaporation of supernatant, reconstituted in 50 µL acidified mobile phase **Cerebrospinal fluid:** 10 µL, 10 µL internal standard solution, 10 µL acidified mobile phase, 150 µL ice-cold MeOH, centrifugation, evaporation of supernatant, reconstituted in 40 µL acidified mobile phase **Brain**: 1-20 mg tissue, 10 µL internal standard, 50 µL acidified mobile phase, 200 µL ice-cold MeOH, homogenization, centrifugation, evaporation of supernatant, reconstituted in 300-500 µL acidified mobile phase  **Alternative method:** ultrafiltration of plasma, brain and CSF samples using membrane cutoff 3 kDa | **Reversed-phase gradient separation:** GRACE VisionHT C18 column (100 x 2.1 mm, 3 µm); **Eluent:** A: 0.01% TFA in water, B: 0.01% TFA in ACN; **Flow rate:** 0.4 mL/min; **Run time:** 5.5 min; **Injection volume:** 20 µL;  **Column temperature:** 15 ^o^C | targeted MS/MS detection,  positive ESI MRM mode | LLOD values were determined in matrix-free standards after the sample preparation: Trp (2.5 nM), KYN (0.5 nM), 3-HK (0.75 nM), 3-HAA (1 nM), QA (2.5 nM), PA (10 nM), KYNA (0.25 nM), XA (0.25 nM) | ([Fuertig et al., 2016](#_ENREF_6)) |
| UHPLC-MS/MS | human serum and cerebrospinal fluid | 100 µL serum or cerebrospinal fluid, 10 µL internal standard solution, 390 µL cooled MeOH, vortexed, incubation (-20 ^o^C, 60 min), centrifugation (micro-spin centrifuge filter tube), evaporation of supernatant, reconstituted in 30 µL water/MeOH (98/2, water/MeOH) using ultrasonication **For TRP and KYN**, 20 times dilution of serum | **Reversed-phase gradient separation:** Acquity UPLC HSS T3 column (100 x 2.1 mm, 1.8 µm); **Eluent:** A: 0.1 % FA in water, B: MeOH; **Flow rate:** 0.30 mL/min; **Run time:** 10 min; **Injection volume:** 10 µL;  **Column temperature:** 30 ^o^C. | targeted MS/MS detection,  positive ESI MRM mode | LLOD values were determined.  **Serum:** Trp (6.00 nM), KYN (0.10 nM), KYNA (0.20 nM), 3-HK (0.15 nM), 3-HAA (0.30 nM) **Cerebrospinal fluid:** Trp (27.0 nM), KYN (0.15 nM), KYNA (0.15 nM), 3-HK (0.03 nM), 3-HAA (0.15 nM) | ([Henykova et al., 2016](#_ENREF_7)) |
| UHPLC-MS/MS | human plasma (EDTA) | 60 µL plasma, 10 μL of the internal standard, 140 µL ACN, centrifugation, evaporation of 180 µL of supernatant, reconstituted in 60 µL water | **Reversed-phase gradient separation:** YMC Triart PFP column (100 x 2.1 mm, 1.9 µm); **Eluent:** A: 0.35% FA in water, B: 95/5, v/v ACN/water; **Flow rate:** 0.25 mL/min; **Run time:** 15 min; **Injection volume:** 3 µL; **Column temperature:** 40 ^o^C | targeted MS/MS detection,  positive ESI MRM mode | LLOD values were not determined.  LLOQ values were determined by standard addition.  Trp (2 µM), KYN (200 nM), QA (25 nM), PA (25 nM), KYNA (200 nM), XA (15 nM), AA (10 nM), 3-HK (15 nM), 3-HAA (10 nM) | ([Karakawa et al., 2019](#_ENREF_8)) |

| **LC-MS method** | **Type of samples** | **Main parameters of the sample preparation procedure** | **Main chromatographic conditions** | **Type of MS detection** | **LLOD or LLOQ of TRP and its metabolites** | **Reference** |
| --- | --- | --- | --- | --- | --- | --- |
| UHPLC-HRMS/MS | mice urine, serum, intestinal contents and liver | **Intestinal contents, cecum and liver tissues** *Protocol 1:* 3 mg lyophilized material, internal standard solution, 900 µL MeOH/water (1/1, v/v), homogenization, centrifugation, evaporation of 900 µL supernatant, reconstituted in 100 µL of MeOH/water (1/9, v/v)  *Protocol 2* for PA, QA, TRP:1 mg lyophilized material, internal standard, 500 µL MeOH/water (1/9, v/v), homogenization, centrifugation, supernatant for analysis **Serum samples** *Protocol 1:* 100 µL serum, internal standard solution, 800 µL MeOH, homogenization, centrifugation, evaporation of supernatant, reconstituted in 100 µL MeOH/water (1/9, v/v) *Protocol 2* (TRP): 10 µL serum, internal standard solution, 500 µL MeOH, homogenization, centrifugation, supernatant for analysis **Urine samples** *Protocol 1:* 10 µL urine, internal standard solution, 240 µL water, homogenization, centrifugation, homogenization, centrifugation, supernatant for analysis | **Reversed-phase gradient separation:** Kinetex XB-C18 column (150 x 2.1 mm, 1.7 µm); **Eluent:** A: 0.5% FA in water, B: 0.5% FA in MeOH; **Flow rate:** 0.25 mL/min; **Run time:**15 min; **Injection volume**: 5 or 2 (*Protocol 2*) µL; **Column temperature:** 55 ^o^C | targeted HRMS/MS detection, positive ESI PRM mode | LLOD values were not determined.  LLOQ values were determined in matrix-free solvent:  Trp (3 nM), KYN (0.75 nM), QA (1.125 nM), PA (3.75 nM), KYNA (0.75 nM), XA (0.75 nM), 3-HK (1.5 nM), 3-HAA (0.1 nM) | ([Lefevre et al., 2019](#_ENREF_9)) |
| LC-MS/MS  based on  Midttun et al., 2009 | human plasma (EDTA) | - | - | - | - | ([Lund et al., 2020](#_ENREF_10)) |
| HPLC-MS/MS | human plasma (EDTA) | 60 µL plasma, 60 µL of 60 g/L trichloroacetic acid (containing the internal standards), centrifugation, collection of 60 µL supernatant for analysis | **Reversed-phase gradient separation:** Zorbax stable-bond C18 column (150 x 4.6 mm, 3.5 µm); **Eluent:** A: 650 nM acetic acid in water, B: 100 nM HFBA in A eluent, C: ACN/water (90/10, v/v);  **Flow rate:** 0.3 mL/min; **Run time:** 6.4 min; **Injection volume:** 50 mL; **Column temperature:** 40 ^o^C | targeted MS/MS detection,  positive ESI MRM mode | LLOD values were determined in 4% albumin in PBS solution:  Trp (400 nM or 4 nM), KYN (7 nM), KYNA (0.4 nM), XA (0.5 nM), 3-HAA (2 nM), 3-HK (2 nM), AA (0.7 nM) | ([Midttun et al., 2009](#_ENREF_11)) |
| GC-MS/MS  HPLC-MS/MS based on  Midttun et al., 2009 | human plasma (EDTA) | - | - | - | - | ([Midttun et al., 2014](#_ENREF_12)) |
| HPLC-MS/MS | rat plasma (EDTA) | SPE, 800 µL plasma, 50 µL internal standard, SPE utilizing 3 cc Waters HLB cartridge, evaporation of supernatant, reconstituted in 150 µL 1 % ammonia solution | **Reversed-phase gradient separation:** Restek C18 Aqueous column (100 x 2.1 mm); **Eluent:** A: ammonium formate in water (0.05% v/v, pH adjusted to 5.5 with acetic acid), B: ACN;  **Flow rate:** 0.2 mL/min; **Run time:** 12 min; **Injection volume:** 10 µL;  **Column temperature:** 25 ^o^C | targeted MS/MS detection,  positive and negative ESI MRM mode | LLOD values: were determined in metabolite-free plasma after the sample preparation.  Trp (17.6 nM), KYN (17.3 nM), KYNA (17.5 nM), AA (23.5 nM), 3-HAA (42.0 nM) QA* (48.2 nM), PA* (1.007 µM)  * detection in negative ESI mode | ([Moller et al., 2012](#_ENREF_13)) |
| HPLC-MS/MS based on Midttun et al., 2009 | human plasma (EDTA) | - | - | - | - | ([Pedersen et al., 2011](#_ENREF_14)) |
| **LC-MS method** | **Type of samples** | **Main parameters of the sample preparation procedure** | **Main chromatographic conditions** | **Type of MS detection** | **LLOD or LLOQ of TRP and its metabolites** | **Reference** |
| GC-MS/MS (urinary metabolites)  HPLC-MS/MS based on Midttun et al., 2009 | human plasma (EDTA), urine | - | - | - | LLOD values: Trp (0.5 mM), KYN (0.1 mM), 3-HK (5 nM) in urine | ([Pedersen et al., 2013](#_ENREF_15)) |
| UHPLC-MS/MS | human serum and peritoneal fluid | **Serum:** 95 µL serum , 5 µL internal standard solution, 50 µL 15 % (w/v) TCA solution, vortexing, centrifugation, supernatant for analysis **For TRP:** 47.5 µL serum , 2.5 µL internal standard solution, 25 µL 15 % (w/v) TCA solution, vortexing, centrifugation, 100 times dilutation of supernatant **Peritoneal fluid:** 395 µL peritoneal fluid, 5 µL internal standard solution, 500 µL cold 1 % FA, vortexing, centrifugation, SPE of supernatant | **Reversed-phase gradient separation:** Zorbax Eclipse Plus C18 Rapid resolution HT column (100 x 2.1 mm, 1.8 µm); **Eluent:** A: 5 mM aqueous ammonium acetate, B: MeOH;  **Flow rate:** 0.25 mL/min; **Run time:** 14 min (for serum), 12 min (for peritoneal fluid); **Injection volume:** 10 µL; **Column temperature:** 40 ^o^C | targeted MS/MS detection,  positive ESI MRM mode | LLOD values were determined in metabolite-free serum.  Trp (0.81 nM), KYN (1.58 nM), QA (3.95 nM), KYNA (1.75 nM), 3-HK (7.59 nM), 3-HAA (-), XA (3.23 nM), AA (12.03 nM)  LLOD values were determined in metabolite-free peritoneal fluid.  Trp (0.81 nM), KYN (1.58), QA (5.92 nM), KYNA (1.76 nM), 3-HK (7.591 nM), 3-HAA (10.77 nM), XA (4.70 nM), AA (12.03 nM) | ([Sadok et al., 2021](#_ENREF_16)) |
| UHPLC-MS/MS | cerebrospinal fluid | 30 µL sample, 30 µL 5 % FA containing internal standard | **Reversed-phase gradient separation:** UPLC HSS T3 column (150 x 2.1 mm, 1.8 µm); **Eluent:** A: 0.6% FA, B: 0.6% FA in MeOH; **Flow rate:** 0.3 mL/min; **Run time:** 13 min; **Injection volume:** 3 µL; **Column temperature:** 50 ^o^C | targeted MS/MS detection,  positive ESI MRM mode | LLOD values were not determined.in cerebrospinal fluid.  Trp (10 nM), KYN (0.1 nM), QA (2.5 nM), KYNA (0.1 nM), 3-HK (0.25 nM), 3-HAA (1 nM) | ([Schwieler et al., 2020](#_ENREF_17)) |
| HPLC-MS/MS | human plasma (EDTA) | 100 µL plasma, 50 µL internal standard, 350 µL 0,1% FA in ACN, centrifugation, evaporation of 300 µl supernatant, reconstituted in 100 µL 0.1% FA, centrifugation, supernatant for analysis | **Reversed-phase gradient separation:** Ultimate XB‐C18 HPLC column (50 x 2.1 mm, 5 µm); **Eluent:** A: 0.1% FA in water, B: 0.1% FA in ACN;  **Flow rate:** 0.35-2.5 mL/min; **Run time:** 3.5 min; **Injection volume:** 5 µL; **Column temperature:**  40 ^o^C | targeted MS/MS detection,  positive ESI MRM mode | LLOD values were not determined.  LLOQ values were determined in matrix-free solvent:  Trp (40 ng/mL), KYN (4 ng/mL), KYNA (0.2 ng/mL), XA (0.4 ng/mL) | ([Tong et al., 2018](#_ENREF_19)) |
| UHPLC-HRMS/MS | human serum, cerebrospinal fluid | **Cerebrospinal fluid** 250 µL cerebrospinal fluid , 20 µL 0.1% FA , 910 µL ice-cold ACN (containing internal standard), incubation (15 min, -20 ^o^C), centrifugation, 1120 µl supernatant splitted into two equal parts, evaporation *first part:* 70 µL derivatizing (n-butanol-acetyl chloride, 9:1, v/v), incubation (60 min, 60 ^o^C), evaporation of reagent, reconstituted in 100 µL starting eluent *second part:* reconstituted in 100 µL starting eluent, both parts combined **Serum** 100 µL serum , 10 µL 0.1% 5 FA , 370 µL ice-cold 1/1 v/v ACN/MeOH (containing internal standard), incubation (15 min, -20 ^o^C), centrifugation, 400 µl supernatant splitted into two equal parts, evaporation, same as above | **Reversed-phase gradient separation:** Kinetex pentafluorophenyl column (100 x 2.6 mm, 2.6 µm); **Eluent:** A: 0.1% FA, B: 0.1% FA in MeOH,  **Flow rate:** 0.3 mL/min; **Run time:** 7 min; **Injection volume**: 20 µL; **Column temperature:** 25 ^o^C | targeted HRMMS/MS detection,  positive ESI PRM mode | LLOD values were determined in metabolite-free cerebrospinal fluid:  Trp (2.724 nM), KYN (1.622 nM), QA (1.236 nM), KYNA (0.083 nM), 3-HK (0.378 nM), 3-HAA (0.094 nM), XA (0.036 nM), PA (0.105 nM), AA (0.168 nM)   LLOQ values were determined in metabolite-free serum.  Trp (1.224 nM), KYN (0.723), QA (6.794 nM), KYNA (0.156 nM), 3-HK (0.601 nM), 3-HAA (1.660 nM), XA (0.073 nM), PA (1.226 nM), AA (1.209 nM) | ([Tomosi et al., 2020](#_ENREF_18)) |
| **LC-MS method** | **Type of samples** | **Main parameters of the sample preparation procedure** | **Main chromatographic conditions** | **Type of MS detection** | **LLOD or LLOQ of TRP and its metabolites** | **Reference** |
| UHPLC-HRMS | human urine | 200 µL urine, 600 µL ACN, vortexed, incubation (60 min, -20 ^o^C), centrifugation, 600 µL supernatant for analysis | **Reversed-phase gradient separation:** UPLC HSS T3 column (100 x 2.1 mm, 1.8 µm); **Eluent:** A: 0.1% FA in water, B: 0.1% FA in ACN; **Flow rate:** 0.5 mL/min; **Run time:** 17 min; **Injection volume:** 20 µL; **Column temperature:** 40 ^o^C. | non targeted HRMS detection,  positive ESI full scan mode | - | ([Wang et al., 2018](#_ENREF_20)) |
| UHPLC-MS/MS | human plasma (EDTA), serum | SPE, 30 µL sample, 10 μL of the internal standard solution, 96- well Phenomenex PHREE cartridge, centrifugation, PHREE SPE plate, centrifugation, evaporation of extract, reconstituted in 40 µL water containing 10 mM ammonium formate and 0.5% formic acid | **TRP, KYN**: **Reversed-phase gradient separation:** UPLC HSS T3 column (150 x 2.1 mm, 1.8 µm); **Eluent:** A: 0.1% FA in water, B: 0.1% FA in ACN; **Flow rate:** 0.3 mL/min; **Run time:** 5 min; **Injection volume:** 5 µL; **Column temperature:** 45 ^o^C | targeted MS/MS detection,  positive and negative ESI MRM mode | LLOD values were not determined.  LLOQ values: Trp* (200 ng/mL), KYN (8 ng/mL), QA (1ng/mL), KYNA (0.4 ng/mL), 3-HK (2 ng/mL), 3-HAA (2 ng/mL), XA (0.4 ng/mL), PA (0.4 ng/mL) * detection in negative ESI mode | ([Whiley et al., 2019](#_ENREF_21)) |
| UHPLC-MS/MS | human serum | 5 µL serum, MeOH, internal standard, vortexed, incubation (-20 ^o^C, 60 min), centrifugation, evaporation of supernatant, reconstituted in borate puffer (pH 8.5) with AQC reagent | **Reversed-phase gradient separation:** Acquity Accq-Tag Ultra column, **Injection volume:** 1 µL | targeted MS/MS detection,  positive ESI MRM mode | - | ([Zhang et al., 2016](#_ENREF_22)) |

Anesi, A., Rubert, J., Oluwagbemigun, K., Orozco-Ruiz, X., Nothlings, U., Breteler, M.M.B., et al. (2019). Metabolic profiling of human plasma and urine, targeting tryptophan, tyrosine and branched chain amino acid pathways. *Metabolites* 9(11). doi: 10.3390/metabo9110261.

Boulet, L., Faure, P., Flore, P., Monteremal, J., and Ducros, V. (2017). Simultaneous determination of tryptophan and 8 metabolites in human plasma by liquid chromatography/tandem mass spectrometry. *J Chromatogr B Analyt Technol Biomed Life Sci* 1054**,** 36-43. doi: 10.1016/j.jchromb.2017.04.010.

Cason, C.A., Dolan, K.T., Sharma, G., Tao, M., Kulkarni, R., Helenowski, I.B., et al. (2018). Plasma microbiome-modulated indole- and phenyl-derived metabolites associate with advanced atherosclerosis and postoperative outcomes. *Journal of Vascular Surgery* 68(5)**,** 1552-1562.e1557. doi: 10.1016/j.jvs.2017.09.029.

de Jong, W.H., Smit, R., Bakker, S.J., de Vries, E.G., and Kema, I.P. (2009). Plasma tryptophan, kynurenine and 3-hydroxykynurenine measurement using automated on-line solid-phase extraction HPLC-tandem mass spectrometry. *J Chromatogr B Analyt Technol Biomed Life Sci* 877(7)**,** 603-609. doi: 10.1016/j.jchromb.2009.01.015.

Eussen, S.J.P.M., Ueland, P.M., Vollset, S.E., Nygård, O., Midttun, Ø., Sulo, G., et al. (2015). Kynurenines as predictors of acute coronary events in the Hordaland Health Study. *International Journal of Cardiology* 189**,** 18-24. doi: 10.1016/j.ijcard.2015.03.413.

Fuertig, R., Ceci, A., Camus, S.M., Bezard, E., Luippold, A.H., and Hengerer, B. (2016). LC-MS/MS-based quantification of kynurenine metabolites, tryptophan, monoamines and neopterin in plasma, cerebrospinal fluid and brain. *Bioanalysis* 8(18)**,** 1903-1917. doi: 10.4155/bio-2016-0111.

Henykova, E., Vranova, H.P., Amakorova, P., Pospisil, T., Zukauskaite, A., Vlckova, M., et al. (2016). Stable isotope dilution ultra-high performance liquid chromatography-tandem mass spectrometry quantitative profiling of tryptophan-related neuroactive substances in human serum and cerebrospinal fluid. *J Chromatogr A* 1437**,** 145-157. doi: 10.1016/j.chroma.2016.02.009.

Karakawa, S., Nishimoto, R., Harada, M., Arashida, N., and Nakayama, A. (2019). Simultaneous analysis of tryptophan and its metabolites in human plasma using liquid chromatography–electrospray ionization tandem mass spectrometry. *CHROMATOGRAPHY* 40(3)**,** 127-133. doi: 10.15583/jpchrom.2019.010.

Lefevre, A., Mavel, S., Nadal-Desbarats, L., Galineau, L., Attucci, S., Dufour, D., et al. (2019). Validation of a global quantitative analysis methodology of tryptophan metabolites in mice using LC-MS. *Talanta* 195**,** 593-598. doi: 10.1016/j.talanta.2018.11.094.

Lund, A., Nordrehaug, J.E., Slettom, G., Hafstad Solvang, S.-E., Ringdal Pedersen, E.K., Midttun, Ø., et al. (2020). Plasma kynurenines and prognosis in patients with heart failure. *PLoS ONE* 15(1). doi: 10.1371/journal.pone.0227365.

Midttun, O., Hustad, S., and Ueland, P.M. (2009). Quantitative profiling of biomarkers related to B-vitamin status, tryptophan metabolism and inflammation in human plasma by liquid chromatography/tandem mass spectrometry. *Rapid Commun Mass Spectrom* 23(9)**,** 1371-1379. doi: 10.1002/rcm.4013.

Midttun, O., Townsend, M.K., Nygård, O., Tworoger, S.S., Brennan, P., Johansson, M., et al. (2014). Most blood biomarkers related to vitamin status, one-carbon metabolism, and the kynurenine pathway show adequate preanalytical stability and within-person reproducibility to allow assessment of exposure or nutritional status in healthy women and cardiovascular patients. *The Journal of Nutrition* 144(5)**,** 784-790. doi: 10.3945/jn.113.189738.

Moller, M., Du Preez, J.L., and Harvey, B.H. (2012). Development and validation of a single analytical method for the determination of tryptophan, and its kynurenine metabolites in rat plasma. *J Chromatogr B Analyt Technol Biomed Life Sci* 898**,** 121-129. doi: 10.1016/j.jchromb.2012.04.030.

Pedersen, E.R., Midttun, Ø., Ueland, P.M., Schartum-Hansen, H., Seifert, R., Igland, J., et al. (2011). Systemic markers of interferon-γ–mediated immune activation and long-term prognosis in patients with stable coronary artery disease. *Arteriosclerosis, Thrombosis, and Vascular Biology* 31(3)**,** 698-704. doi: 10.1161/ATVBAHA.110.219329.

Pedersen, E.R., Svingen, G.F.T., Schartum-Hansen, H., Ueland, P.M., Ebbing, M., Nordrehaug, J.E., et al. (2013). Urinary excretion of kynurenine and tryptophan, cardiovascular events, and mortality after elective coronary angiography. *European Heart Journal* 34(34)**,** 2689-2696. doi: 10.1093/eurheartj/eht264.

Sadok, I., Jedruchniewicz, K., Rawicz-Pruszynski, K., and Staniszewska, M. (2021). UHPLC-ESI-MS/MS Quantification of Relevant Substrates and Metabolites of the Kynurenine Pathway Present in Serum and Peritoneal Fluid from Gastric Cancer Patients-Method Development and Validation. *Int J Mol Sci* 22(13). doi: 10.3390/ijms22136972.

Schwieler, L., Trepci, A., Krzyzanowski, S., Hermansson, S., Granqvist, M., Piehl, F., et al. (2020). A novel, robust method for quantification of multiple kynurenine pathway metabolites in the cerebrospinal fluid. *Bioanalysis* 12(6)**,** 379-392. doi: 10.4155/bio-2019-0303.

**References:**

Tomosi, F., Kecskemeti, G., Cseh, E.K., Szabo, E., Rajda, C., Kormany, R., et al. (2020). A validated UHPLC-MS method for tryptophan metabolites: Application in the diagnosis of multiple sclerosis. *J Pharm Biomed Anal* 185**,** 113246. doi: 10.1016/j.jpba.2020.113246.

Tong, Q., Song, J., Yang, G., Fan, L., Xiong, W., and Fang, J. (2018). Simultaneous determination of tryptophan, kynurenine, kynurenic acid, xanthurenic acid and 5-hydroxytryptamine in human plasma by LC-MS/MS and its application to acute myocardial infarction monitoring. *Biomedical chromatography: BMC* 32(4). doi: 10.1002/bmc.4156.

Wang, Y., Sun, W., Zheng, J., Xu, C., Wang, X., Li, T., et al. (2018). Urinary metabonomic study of patients with acute coronary syndrome using UPLC-QTOF/MS. *Journal of Chromatography. B, Analytical Technologies in the Biomedical and Life Sciences* 1100-1101**,** 122-130. doi: 10.1016/j.jchromb.2018.10.005.

Whiley, L., Nye, L.C., Grant, I., Andreas, N., Chappell, K.E., Sarafian, M.H., et al. (2019). Ultrahigh-performance liquid chromatography tandem mass spectrometry with electrospray ionization quantification of tryptophan metabolites and markers of gut health in serum and plasma-application to clinical and epidemiology cohorts. *Anal Chem* 91(8)**,** 5207-5216. doi: 10.1021/acs.analchem.8b05884.

Zhang, Y., Blasco-Colmenares, E., Harms, A.C., London, B., Halder, I., Singh, M., et al. (2016). Serum amine-based metabolites and their association with outcomes in primary prevention implantable cardioverter-defibrillator patients. *Europace: European Pacing, Arrhythmias, and Cardiac Electrophysiology: Journal of the Working Groups on Cardiac Pacing, Arrhythmias, and Cardiac Cellular Electrophysiology of the European Society of Cardiology* 18(9)**,** 1383-1390. doi: 10.1093/europace/euv342.
